# A spatially resolved timeline of the human maternal–fetal interface

**DOI:** 10.1038/s41586-023-06298-9

**Published:** 2023-07-19

**Authors:** Shirley Greenbaum, Inna Averbukh, Erin Soon, Gabrielle Rizzuto, Alex Baranski, Noah F. Greenwald, Adam Kagel, Marc Bosse, Eleni G. Jaswa, Zumana Khair, Shirley Kwok, Shiri Warshawsky, Hadeesha Piyadasa, Mako Goldston, Angie Spence, Geneva Miller, Morgan Schwartz, Will Graf, David Van Valen, Virginia D. Winn, Travis Hollmann, Leeat Keren, Matt van de Rijn, Michael Angelo

**Affiliations:** 1 Department of Pathology, Stanford University, Stanford, CA USA; 2 Department of Obstetrics and Gynecology, Hadassah-Hebrew University Medical Center, Jerusalem, Israel; 3 Immunology Program, Stanford University, Stanford, CA USA; 4 Department of Pathology, University of Californica San Francisco, San Francisco, CA USA; 5 Cancer Biology Program, Stanford University, Stanford, CA USA; 6 Department of Obstetrics Gynecology and Reproductive Sciences, University of California San Francisco, San Francisco, CA USA; 7 Division of Biology and Bioengineering, California Institute of Technology, Pasadena, CA USA; 8 Department of Obstetrics and Gynecology, Stanford University, Stanford, CA USA; 9 Department of Pathology, Memorial Sloan Kettering Cancer Center, New York, NY USA; 10 Department of Molecular Cell Biology, Weizmann Institute of Science, Rehovot, Israel

**Keywords:** Immunology, Developmental biology, Reproductive biology

## Abstract

Beginning in the first trimester, fetally derived extravillous trophoblasts (EVTs) invade the uterus and remodel its spiral arteries, transforming them into large, dilated blood vessels. Several mechanisms have been proposed to explain how EVTs coordinate with the maternal decidua to promote a tissue microenvironment conducive to spiral artery remodelling (SAR)^1–3^. However, it remains a matter of debate regarding which immune and stromal cells participate in these interactions and how this evolves with respect to gestational age. Here we used a multiomics approach, combining the strengths of spatial proteomics and transcriptomics, to construct a spatiotemporal atlas of the human maternal–fetal interface in the first half of pregnancy. We used multiplexed ion beam imaging by time-of-flight and a 37-plex antibody panel to analyse around 500,000 cells and 588 arteries within intact decidua from 66 individuals between 6 and 20 weeks of gestation, integrating this dataset with co-registered transcriptomics profiles. Gestational age substantially influenced the frequency of maternal immune and stromal cells, with tolerogenic subsets expressing CD206, CD163, TIM-3, galectin-9 and IDO-1 becoming increasingly enriched and colocalized at later time points. By contrast, SAR progression preferentially correlated with EVT invasion and was transcriptionally defined by 78 gene ontology pathways exhibiting distinct monotonic and biphasic trends. Last, we developed an integrated model of SAR whereby invasion is accompanied by the upregulation of pro-angiogenic, immunoregulatory EVT programmes that promote interactions with the vascular endothelium while avoiding the activation of maternal immune cells.

## Abstract (web summary)

A multiomics approach is used to produce a spatiotemporal atlas of the human maternal–fetal interface in the first half of pregnancy, revealing relationships among gestational age, extravillous trophoblasts and spiral artery remodelling.

## Main

Normal development during healthy pregnancy depends on a complex interplay between maternal cells and placental trophoblasts that ultimately transforms the womb into a specialized niche capable of meeting the metabolic demands of a growing semi-allogeneic fetus while maintaining maternal tolerance^1,2^. After implantation, the decidua is invaded by EVTs. EVTs and maternal cells remodel uterine spiral arteries into highly dilated vessels with minimal smooth muscle where EVTs have partially replaced the maternal endothelium^3^. In healthy pregnancies, SAR results in low-resistance vessels that deliver blood to the intervillous space at low flow velocities that prevent damage to the placental architecture^4^. Conversely, impaired SAR, low numbers of tolerogenic maternal cells and abnormal decidual invasion of EVTs have each been implicated in placenta-related obstetric complications, including preeclampsia, intrauterine growth restriction and preterm birth^5^. Therefore, a detailed investigation of the cell population dynamics at the maternal–fetal interface is key to understanding the biology of normal pregnancy and obstetric complications.

Owing to the poor feasibility of controlled studies in pregnant humans, much of what is known about maternal–fetal tolerance and SAR is based on small-animal models^6^. Although some similarities exist, key facets of haemochorial placentation in humans are primate-specific^7^. For example, EVT giant cells in mice do not replace the vascular endothelium and are thought to play a minor part in SAR compared to maternal uterine natural killer (NK) cells^8^. The extensive degree of EVT invasion in humans is thought to be an evolutionary adaptation that permitted upright, bipedal locomotion while maintaining adequate blood flow in the third trimester when brain development accounts for 60% of fetal metabolic needs^9^.

The study of human decidual remodelling is further complicated by additional inherent challenges. First, cell composition and structure are temporally dynamic. Therefore, aggregating data across different gestational ages (GAs) or observing a single time point may be misleading. Second, these dynamics are spatially coordinated in the local tissue microenvironment. For example, periarterial decidual NK cells are thought to contribute to SAR by initiating smooth muscle breakdown and by secreting chemokines that attract invading EVTs, whereas phagocytic macrophages are thought to facilitate clearance of the resultant apoptotic debris^10^. Overall, the formation of the human maternal–fetal interface involves sophisticated spatiotemporal coordination such that tissue composition, structure and function are inextricably coupled.

With this in mind, we constructed a multimodal spatiotemporal atlas of the human maternal–fetal interface. We leveraged archival tissue banks to assemble a cohort of maternal decidua from 66 women who underwent elective terminations of otherwise healthy pregnancies at 6–20 weeks of gestation, constituting a large single-cell study of the maternal–fetal interface. We performed subcellular imaging with multiplexed ion beam imaging by time-of-flight (MIBI-TOF)^11^ using a 37-plex antibody panel designed to identify the location, lineage and function of all major maternal and fetal cells.

We also profiled the transcriptome of arteries, decidua and EVTs. To understand how SAR relates to local decidual composition, we developed new algorithms to quantify vascular morphology that enabled us to assign a remodelling score to each individual artery. We discerned which changes in decidual composition, transcriptome and structure were preferentially driven by GA, SAR or both. Overall, the frequencies and spatial distribution of maternal immune cells exhibited a strong temporal dependence that enabled us to predict GA exclusively on the basis of these features.

By contrast, EVT invasion and perivascular localization were the dominant drivers of SAR in the tissue microenvironment, and these processes correlated with extensive shifts in arterial transcription. Given these findings, we used our atlas to characterize the temporal nature of intravascular EVT invasion in the decidua basalis. The accumulation of perivascular EVTs around arteries preceded smooth muscle loss and the appearance of intravascular EVTs, which is consistent with a model whereby perivascular EVTs intravasate into the artery lumen. Taken together, these investigations support a cooperative interplay in the first half of pregnancy in which temporally dependent changes in decidual function permit placental EVTs to extensively alter the maternal uterine vasculature.

## Multiplexed imaging of the decidua

**Fig. 1:**
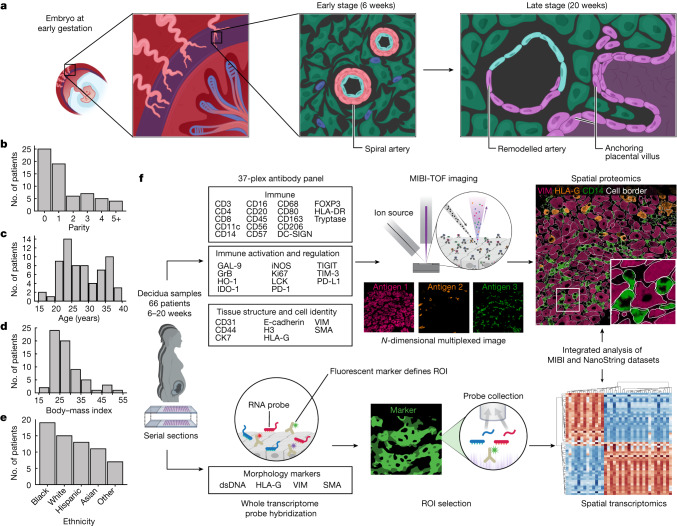
Study design and workflow. **a**, Diagram of a human embryo in utero at 6 weeks of gestation. Left, the maternal–fetal interface consisting of decidua basalis (purple) with maternal spiral arteries (light pink) and fetal chorionic villi in the intervillous space (bottom right corner). Middle and right, early-stage (6 weeks) unremodelled spiral artery and progression to late-stage (20 weeks) remodelled artery and anchoring fetal villi. **b**, Cohort parity distribution. **c**, Cohort age distribution. **d**, Cohort distribution of body–mass index. **e**, Cohort ethnicity distribution. **f**, TMA construction and serial sections for multiomics workflow. Top, antibody panel, MIBI acquisition and spatial proteomics data extraction. Bottom, morphology marker panel and probe diagram, NanoString DSP ROI selection and spatial transcriptomics data extraction. The schematics in **f** were created using BioRender (https://biorender.com). Source Data

As part of the Human BioMolecular Atlas Program initiative, we created a spatiotemporal tissue atlas of the human maternal–fetal interface in the first 20 weeks of pregnancy (Fig. 1a). The goal of this study was to comprehensively define the structure and composition of decidua and to understand how it evolves in the first two trimesters with respect to two axes: GA and maternal SAR. We first assembled a large retrospective cohort of archival formalin-fixed, paraffin-embedded placenta and decidua tissue from 66 individuals who underwent elective termination of pregnancies with no known fetal abnormalities. Archival tissue blocks were manually screened by a perinatal pathologist. Tissue sections stained with haematoxylin and eosin (H&E) were analysed to determine which samples contained decidua, and mostly regions that contained anchoring villi were selected. Then, regions of decidua that contained spiral arteries were demarcated, cored and assembled into two tissue microarrays (TMAs) of 1 mm and 1.5 mm cores. The final dataset included samples for 6–20 weeks of gestation (13.72 ± 4.8 weeks, mean ± SD) from 66 women of varying parity (1.45 ± 1.72), age (28.17 ± 5.9 years), body–mass index (28.19 ± 7.3 kg m^–2^) and ethnicity (Fig. 1b–e and Supplementary Table 1). Owing to inherent limitations in how the tissue was procured, precise anatomical locations could not be determined. However, 61 out of 66 tissue blocks contained placental villi, which suggested that the majority of this cohort was sampled from the decidua basalis (Supplementary Table 1 and Methods).

Previous studies of intact tissue that examined only one or a few cell populations at a time reported shifts in maternal immune cells towards tolerogenic states that are permissive to invasion by fetal EVTs^12^. To gain a more complete picture of the complex cell–cell interactions that establish maternal tolerance in the first half of pregnancy, we combined the strengths of targeted subcellular imaging with antibodies and spatial transcriptomics on serial co-registered sections to construct a comprehensive composite model of SAR and decidual remodelling (Fig. 1f).

For MIBI-TOF, we designed and validated a 37-plex antibody panel to map the functional state and location of all major maternal and fetal cell populations (Fig. 1f and Methods). This panel included canonical lineage-defining markers and ten functional markers previously implicated in maternal immune tolerance^13–15^ (Fig. 1f).

For spatial transcriptomics, we used the NanoString GeoMx Digital Spatial Profiler (DSP) for whole transcriptome analysis of arteries, EVTs and decidua. Immunofluorescence imaging of TMAs stained with antibodies for HLA-G, vimentin (VIM) and smooth muscle actin (SMA) were used to define regions of interest (ROIs) specific for each of these histological features (Methods). In total, we collected whole transcriptome data from 13 individual arteries, their adjacent decidua, 5 samples of interstitial and 3 samples of intravascular EVTs (19 cores from 17 individuals; Methods).

**Fig. 2:**
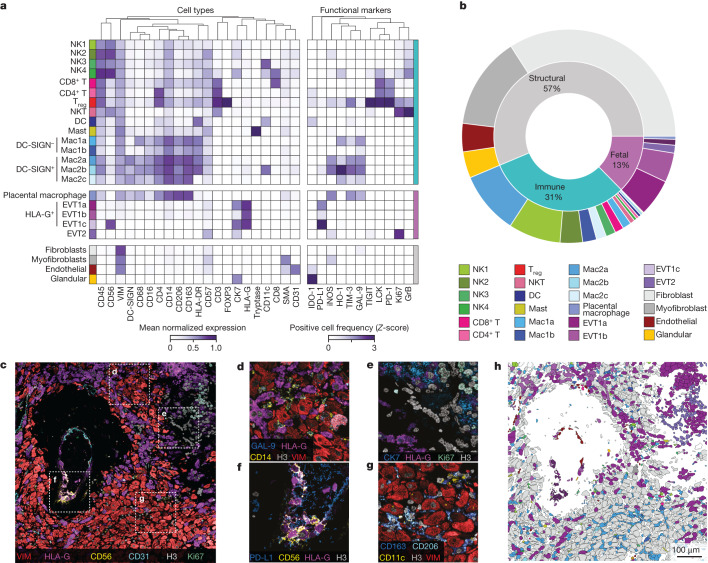
Multiplexed imaging of human decidua reveals the immune-tolerance-conducive composition of the maternal–fetal interface. **a**, Cell lineage assignments showing mean normalized expression of lineage markers (left) and functional-marker-positive cell frequency (right, *Z*-score). Columns (markers) are hierarchically clustered. DC, dendritic cell. **b**, Cell lineage abundances across our cohort. **c**, MIBI field of view (FOV) colour overlay of a 20-week sample. Representative image of *n* = 33 FOVs. **d**, Inset of **c** showing interstitial fetal EVTs. **e**, Inset of **c** showing anchoring villous cell column to decidua interface. **f**, Inset of **c** showing intravascular EVTs. **g**, Inset of **c** showing decidual stromal cells (fibroblasts) and macrophages. **h**, Cell lineage assignments overlaid onto the cell-segmentation output to produce a cell phenotype map. Source Data

**Extended Data Fig. 1:**
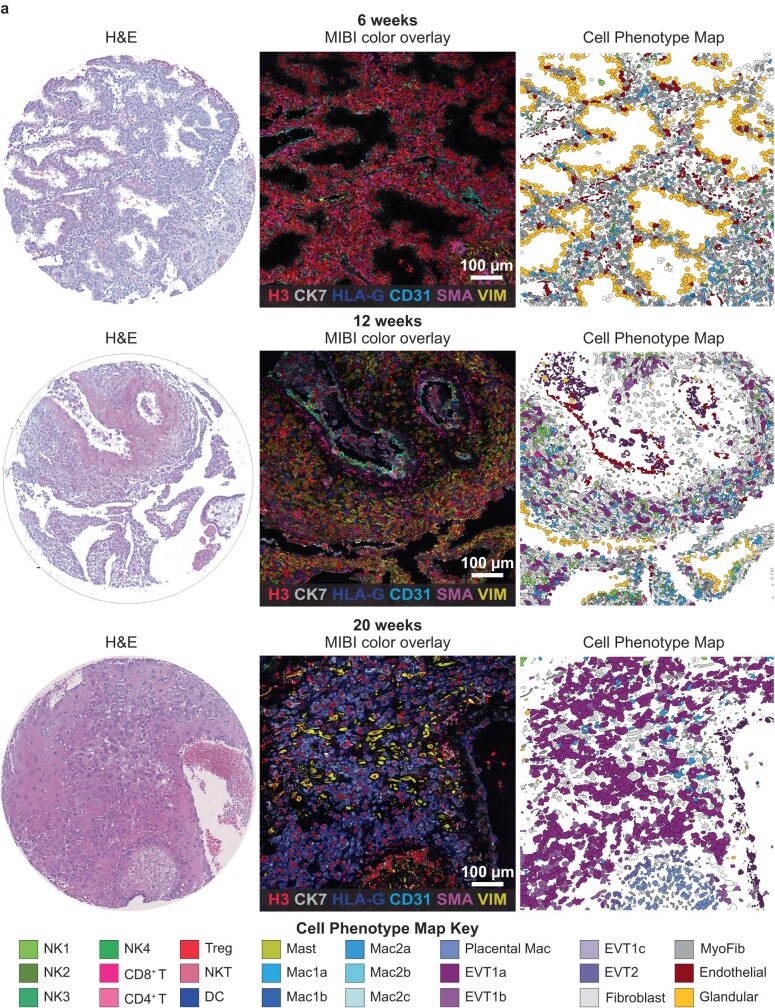
Representative H&E and MIBI images for cell phenotyping. **a**. Serial sections with cell phenotype maps. Each cell colored by its phenotype assignment for GAs: 6, 12, 20 weeks. VIM, vimentin; SMA, smooth muscle actin; Placental Mac, Placental Macrophage; MyoFib, Myofibroblast. Representative images of n = 55 FOVs (6 weeks), n = 12 FOVs (12 weeks), n = 33 FOVs (20 weeks). Scale bar, 100 µm.

For cell segmentation, we used an optimized version of our previously validated deep-learning pipeline that was refined for decidua-specific cell types using 93,000 manual annotations (Methods). In total, we identified 495,349 segmented cells across 211 images and classified them into 25 cell populations (Fig. 2a,b, Methods and Extended Data Fig. 1). Functional marker expression in these populations was determined using per-marker thresholds (Methods). Noteworthy histological features—such as arteries, vessels, glands, the cell columns and decidual tissue boundaries—were manually annotated in collaboration with a perinatal pathologist.

Non-immune maternal (structural) cells accounted for the majority (56.3%) of all segmented events in the decidua and were predominantly composed of decidual fibroblasts (60.5%) and myofibroblasts (24.8%), with smaller contributions from vascular endothelial cells (7.6%) and glandular epithelial cells (7.1%; Fig. 2b). Notably, we observed a new, rare subset of TIGIT^+^ glandular cells (0.34% of glandular cells; Supplementary Information). Consistent with previous work^16^ that quantified maternal populations in situ, maternal immune cells (31% of all cells) were dominated by macrophages (47.6% of immune cells) and NK cells (42.6% of immune cells), with minor contributions from T cells (8% of immune cells), dendritic cells (1.3% of immune cells) and mast cells (0.5% of immune cells).

Decidual macrophages ubiquitously expressed both CD163 and CD206, which is consistent with a M2-polarized, tolerogenic phenotype (Fig. 2g). In line with previous work^17^ that showed pregnancy-specific recruitment, 77% of these cells expressed DC-SIGN (Fig. 2a). We further classified DC-SIGN^+^ macrophages into three subsets on the basis of CD11c and HLA-DR expression: Mac2a, Mac2b and Mac2c. DC-SIGN^–^ macrophages (Mac1a and Mac1b) were subclustered on the basis of CD68 expression (Fig. 2a,b).

**Extended Data Fig. 2:**
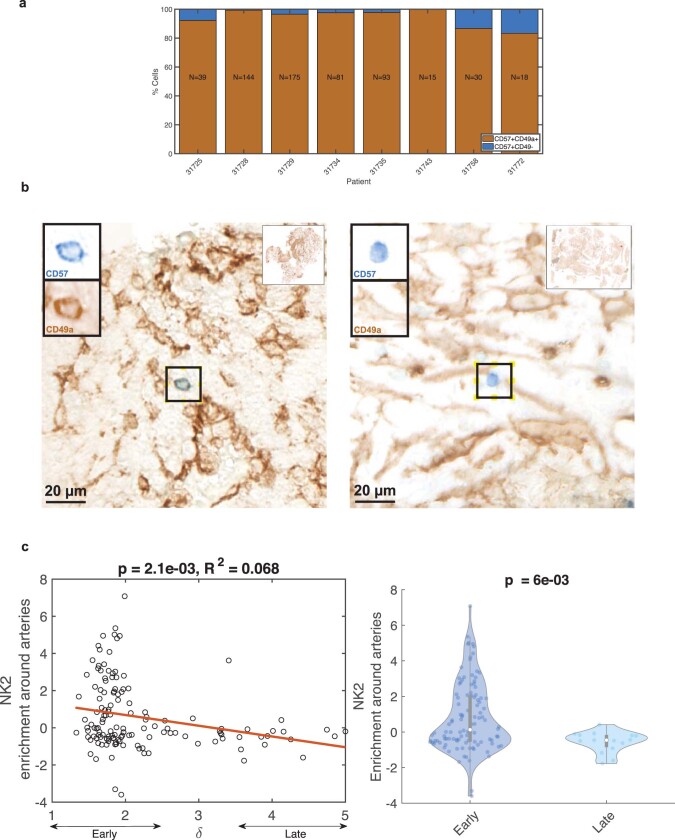
Validation of CD57+ NK2 population. **a**. Bar plot of double positive cells (CD57+CD49a+) percent out of CD57+ cells per patient. **b**. Representative examples of double positive (CD57+CD49a+, left) and single positive (CD57+CD49a-, right) staining using dual color IHC for CD57 and CD49a on decidua samples, 595 cells in total. Representative images of n = 574 double positive cells and n = 21 single positive cells. Scale bar, 20 µm. **c**. Pairwise enrichment between NK2 cells and arteries. Left: mean enrichment per image by δ, n = 209 images. Red line – fitted linear regression, regression p-value. Right: Violin plot of NK2-artery enrichment scores by early (1 ≤ δ < 3) and late (3 ≤ δ < 5) remodeling stage. p-value Kruskal-Wallis. Early: n = 108, min = −3.6, max= 7.07, center = 0.13; Late: n = 15, min = −1.77, max=0.41, center = −0.45. Source Data

Four subsets of NK cells (NK1, NK2, NK3 and NK4) were classified on the basis of the combinatorial expression of CD57, CD11c and CD8. NK1 cells (CD57^–^CD16^low^) constituted the largest NK cell population present, making up 59.7% of NK cells (Fig. 2a,b). A new CD57^+^ population of decidual NK cells (NK2, 25.8% of NK cells) that had only been previously identified in peripheral blood during pregnancy^18^ was also identified. Moreover, most of this population expressed the tissue-residency marker CD49a (94.2%; Extended Data Fig. 2a,b). As described below, the frequency and spatial distribution of NK2 cells suggests that they play a distinct role in SAR (Extended Data Fig. 2c).

T cells consisted of CD8^+^ cells (53.2% of T cells), NK T (NKT) cells (28.8% of T cells), CD4^+^ cells (17.1% of T cells) and sparse numbers of regulatory T (T_reg_) cells (Fig. 2a,b). We identified a PD-1^+^-activated population of T_reg_ cells with a TIM-3^+^LCK^+^ subset that accounted for >50% of this population (Fig. 2a,b). Notably, both T_reg_ cells and NKT cells were the most proliferative cell populations. Together with CD8^+^ NK cells, T_reg_ cells and NKT cells expressed granzyme B more frequently than any other cell type. TIGIT was most frequently expressed by T_reg_ cells—a rare subset that has been suggested to bind PVR (also known as CD155) on EVTs^2^. This interaction has been observed in the tumour microenvironment^19^ and may serve a similar role in driving maternal–fetal tolerance.

Fetal cells (12.6% of all cells) were primarily composed of four subsets of EVTs that were delineated on the basis of the combinatorial expression of HLA-G, CK7, CD57 and CD56 (Fig. 2a). HLA-G^+^ EVTs were CK7^+^ (EVT1a), CK7^–^ (EVT1b) or CD56^+^ (EVT1c) (Fig. 2c–f). EVT2 lacked HLA-G and were CD57^–^CK7^low^ and were located predominantly at the base of attaching cell columns. Taken together, these data provide spatial context to previous work that used dissociated samples^15,20^; that is, an ensemble of functional states in fetal and maternal cells are collectively aligned to maintain a tolerogenic niche.

## SAR correlates with local composition

Perfusion of the intervillous space by uterine spiral arteries is the sole source of oxygen and nutrients to the growing fetus after the establishment of arterial flow. During the first half of pregnancy, these vessels undergo an extensive remodelling process that culminates in dilated, non-contractile vessels depleted of smooth muscle where the maternal endothelium has been partially replaced by EVTs. Abnormal SAR is associated with obstetric complications such as intrauterine growth restriction and preeclampsia^5^. However, it is still not fully understood which cell populations directly participate in SAR, how this process is locally regulated and to what extent these changes are synchronized with GA.

**Fig. 3:**
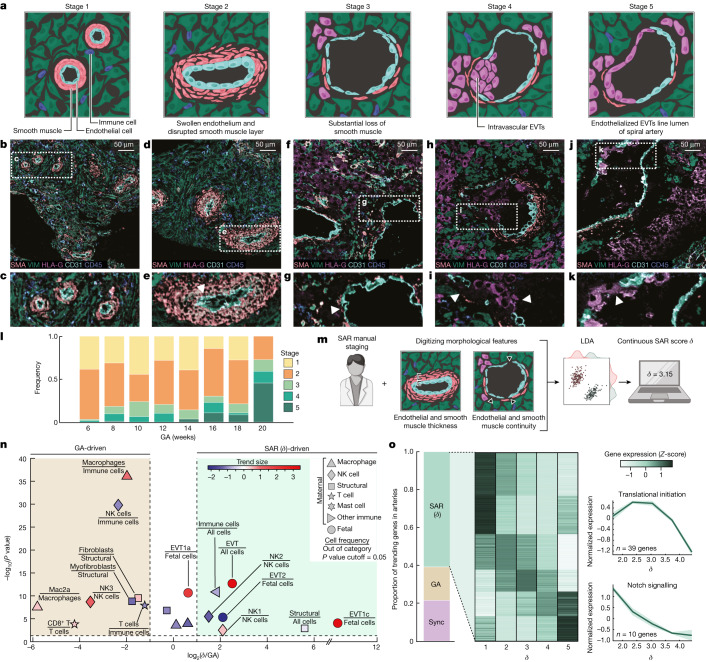
SAR progression significantly influences maternal–fetal interface composition. **a**, Characteristics of SAR stages 1–5 manually assessed. **b**, MIBI colour overlay of manually assessed stage 1 arteries. Representative image of *n* = 70 FOVs. **c**, Inset of **b** showing stage 1 arteries. **d**, MIBI colour overlay of manually assessed stage 2 arteries. Representative image of *n* = 98 FOVs. **e**, Inset of **d**. Arrowhead indicates swollen endothelial cells. **f**, MIBI colour overlay of manually assessed stage 3 arteries. Representative image of *n* = 29 FOVs. **g**, Inset of **f**. Arrowhead indicates substantial loss of smooth muscle. **h**, MIBI colour overlay of one manually assessed stage 4 artery. Representative image of *n* = 21 FOVs. **i**, Inset of **h**. Arrowheads indicate intravascular EVTs. **j**, MIBI colour overlay of one manually assessed stage 5 artery. Representative image of *n* = 20 FOVs. **k**, Inset of **j**. Arrowhead indicates endothelialized EVTs lining the spiral artery lumen. **l**, Distribution of SAR manually assessed stages by GA. GA in days is binned to weeks for visualization. **m**, Schematic of calculating the continuous SAR remodelling score (*δ*). **n**, Volcano plot distinguishing GA-driven from SAR (*δ*)-driven cell-type frequencies. *x* axis, log_2_ ratio of *R*^2^ derived from linear regression against SAR (*δ*) and GA. *y* axis, –log_10_ of the *P* value for the better-fitting regression model. Points are colour-coded by the trend size observed in the better-fitting regression model. **o**, Left, proportion of genes in artery (2,932 in total) tissue where expression changes significantly correlate with SAR (*δ*) (1,785), GA (517) or both (Sync; 633). Centre, SAR (*δ*)-correlated genes in artery tissue showing mean normalized expression (*Z*-score) by SAR (*δ*) stage. Right, two SAR (*δ*)-trending gene ontology pathways showing normalized expression of genes in the GO pathway by SAR (*δ*). Data are presented as the mean gene expression ± s.e.m. Source Data

**Extended Data Fig. 3:**
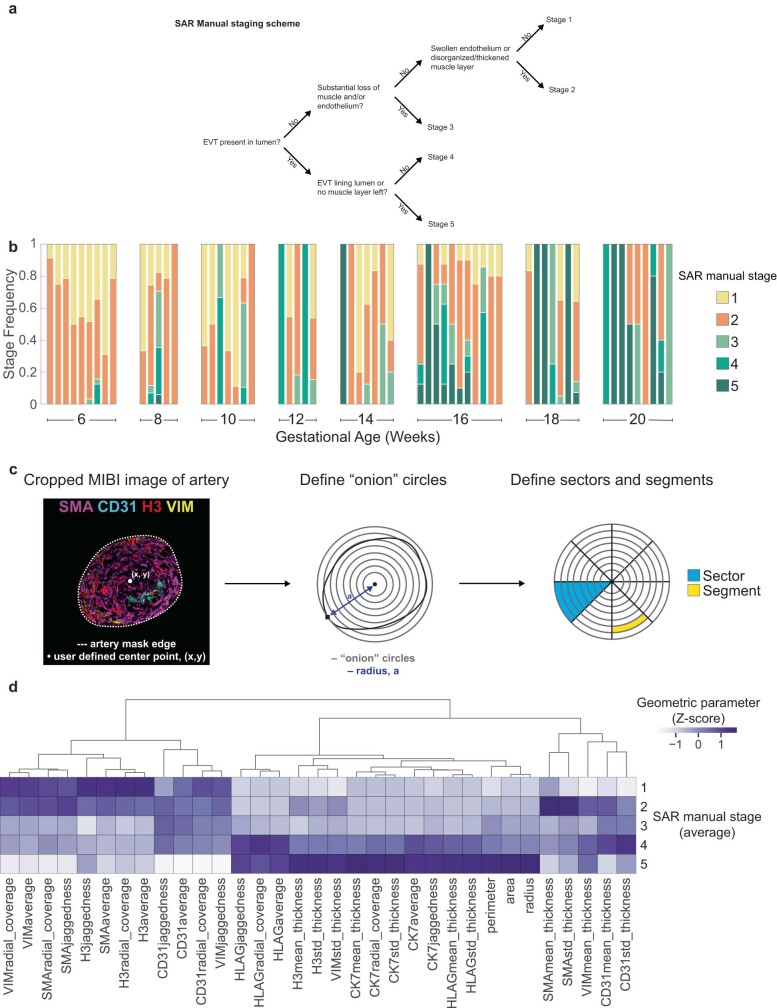
SAR manual and digitized staging. **a**. Flowchart for manual staging of arteries. **b**. Bar plot of manually-determined artery stage frequencies per patient. **c**. Schematic of digitization of artery morphology features. **d**. Heatmap of quantified digitized morphological features, mean across SAR stages. Columns Z-scored and hierarchically clustered. Source Data

We therefore used our spatiotemporal atlas to construct a SAR trajectory to understand how this relates to temporal changes in decidua composition and structure^21^. Using artery size, smooth muscle disruption, endothelial continuity and EVT infiltration, we manually assigned each artery to one of five sequential remodelling stages on the basis of previously published criteria (Methods and Fig. 3a). To ensure that scoring was not biased by demographics of the individuals or the composition of neighbouring arteries and stroma, scoring was performed by blinded experts on cropped images in which only the artery of interest was visible. Out of 588 arteries, 186 were unremodelled and assigned to stage 1 (Fig. 3b,c). Stage 2 arteries (300 arteries) were characterized by moderate smooth muscle disruption and endothelial swelling (Fig. 3d,e). Stage 3 arteries (43 arteries) exhibited more dilation, smooth muscle loss and early endothelial disruption (Fig. 3f,g). Progression to stage 4 (34 arteries) was marked by the presence of EVTs within the arterial lumen (Fig. 3h,i). Fully remodelled stage 5 arteries (25 arteries) were identified on the basis of their very large size, near-complete smooth muscle loss and EVT endothelization (Fig. 3j,k, Extended Data Fig. 3a and Supplementary Table 2).

Although SAR correlated to some extent with GA (Spearman’s *ρ* = 0.28, *P* = 1.5 × 10^−12^), in many cases these were discordant. For example, at least one late-stage artery (stage 4–5) was present in 40% of week 8 samples, whereas minimally remodelled arteries were present throughout (Fig. 3l). Moreover, SAR staging of arteries from the same individual often varied significantly between tissue cores (32% of individuals had arteries that differed by at least two stages), which suggested that aspects of SAR are locally regulated by the tissue microenvironment (Fig. 3l and Extended Data Fig. 3b).

**Extended Data Fig. 4:**
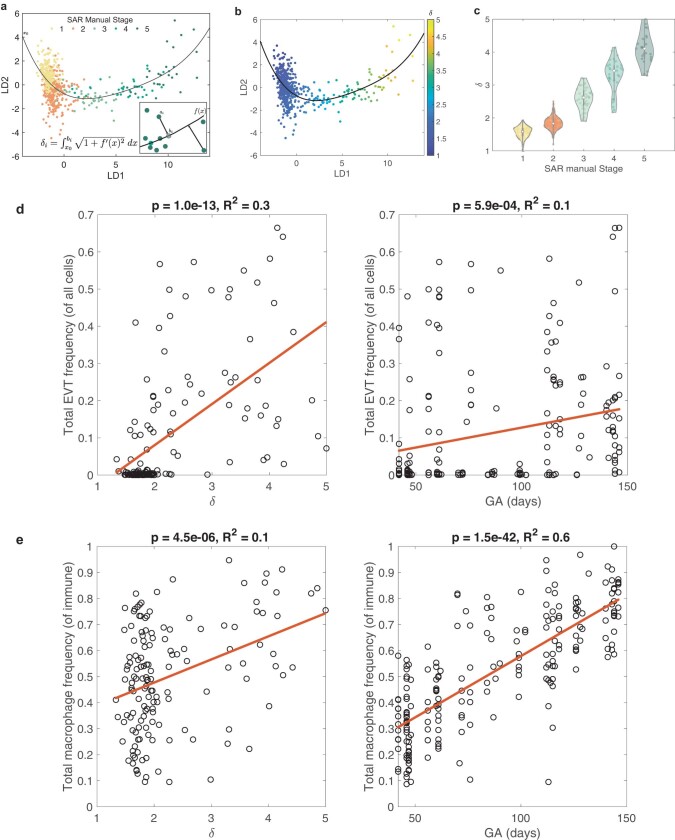
SAR trajectory. **a**. Scatter of arteries in LDA space colored by manually-assigned stage. Polynomial fit: remodeling trajectory. Inset: matching each artery point a_i_ to the SAR trajectory by finding the nearest point along trajectory b_i_. The continuous SAR score δ was then defined as the distance from origin x_0_ to b_i_ along the trajectory curve. **b**. Scatter plot of arteries in LDA space colored by δ. **c**. Violins of artery δs by manual stage **d**,**e**. Scatter plots by δ (left) and GA (right) of EVT frequency out of all cells (**d**), macrophage frequency out of immune cells (**e**) Red lines, fitted linear regression, regression p-values on top. Source Data

We exploited this discordance between SAR and GA to identify changes in decidual composition that were predominantly driven by one or the other. We first developed a quantitative scheme to assign a continuous remodelling score. For each artery, we extracted 35 parameters that described the same aspects of arterial morphology used for manual scoring (Fig. 3m, Methods and Extended Data Fig. 3c,d). Together with manual staging, we used this profile to construct a highly resolved pseudotime trajectory of SAR using linear discriminant analysis (LDA). This trajectory assigned a continuous remodelling score to each artery (*δ*) (Fig. 3m and Methods). Thus, each artery in our dataset could be mapped along a temporal or remodelling trajectory using GA or *δ*, respectively (Extended Data Fig. 4a–c).

Applying linear regression to these values per image, we determined which aspects of decidual remodelling were preferentially correlated with GA or SAR (Methods and Extended Data Fig. 4d,e). The frequency of decidual EVTs was better correlated with SAR, whereas changes in the proportion of maternal immune cells were mostly driven by GA (Fig. 3n). A notable exception to the latter correlation was observed within the NK cell compartment, in which the ratio of NK2 cells (CD57^+^) to NK1 cells (CD57^–^) decreased with SAR progression (Fig. 3n).

To further investigate this finding, we examined how the spatial distribution of NK cells near arteries changed as SAR progressed (Methods). Notably, NK2 cells were the only subset of maternal immune cells to preferentially localize around arteries (Supplementary Table 3). NK2 cell accumulation around arteries spiked specifically at stage 2 of SAR, when smooth muscle swelling and disruption are maximal (*P* =2 × 10^−3^; Extended Data Fig. 2c). Notably, CD57 expression in human NK cells is associated with a cytotoxic phenotype in tumours^18^, which suggests that this subset could serve a similar role in mediating early smooth muscle disruption during SAR.

To create a transcriptional trajectory that integrated with our spatial proteomics data, we used NanoString DSP on serial sections of the TMA imaged by MIBI-TOF. We collected whole transcriptome profiles of 13 individual arteries at various stages of remodelling and their adjacent decidua (a total of 26 ROIs; Methods). Matching these samples with their respective MIBI-TOF images enabled us to assign a remodelling score and GA to each transcriptome profile (Methods). We then used a methodology similar to that presented in Fig. 3n to categorize genes that displayed temporal expression trends as correlated with either SAR, GA or both (Methods).

**Extended Data Fig. 5:**
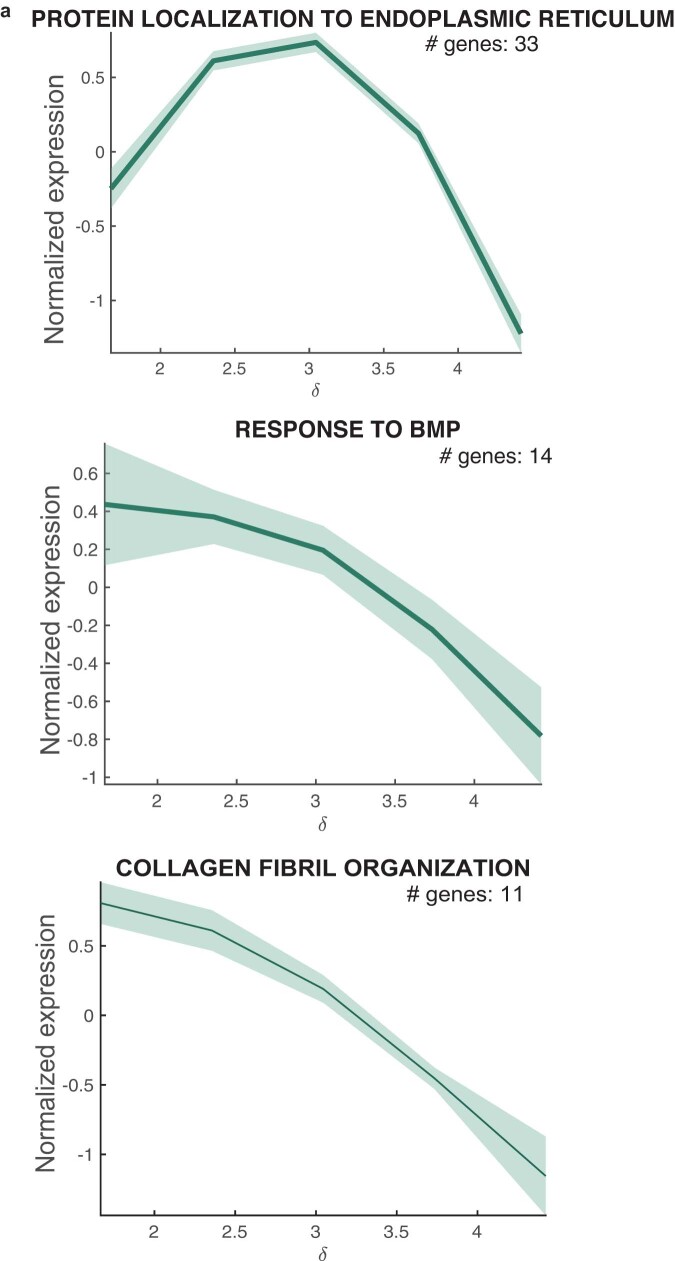
SAR-trending GO pathways. **a**. 3 SAR (δ)-trending GO pathways, showing normalized expression of genes in the pathway by SAR (δ): mean over genes +/− SEM. Source Data

For arteries, 2,935 out of 18,695 genes exhibited significant trends, with most genes preferentially correlating with SAR (Fig. 3o). Within this group of genes, we identified 78 temporally synchronized gene ontology pathways, including modules related to vessel remodelling and translation (Methods and Supplementary Table 4). These pathways exhibited both monotonic and biphasic trends (Fig. 3o), which showed that SAR is a composite of interrelated processes that occur continuously and episodically. We identified 185 genes that peaked at stage 2 of remodelling before subsequently declining. This expression pattern correlated with perivascular enrichment of NK2 cells as indicated by our MIBI data (Extended Data Fig. 2c). In addition, this group of genes was enriched for genes related to collagen fibril organization and responses to bone morphogenic protein (Fig. 3o and Extended Data Fig. 5). Consistent with cell growth and subsequent apoptosis of arterial smooth muscle, translation-related genes followed a biphasic trend, peaking at around stage 3 of remodelling (Fig. 3o and Extended Data Fig. 5). We also observed continual downregulation of genes involved in Notch signalling as SAR progressed (Fig. 3o). Taken together, these multimodal data provide a fully integrated atlas of decidual remodelling that describes tissue structure, single-cell function and changes in transcriptional programmes.

## Immune composition correlates with GA

**Fig. 4:**
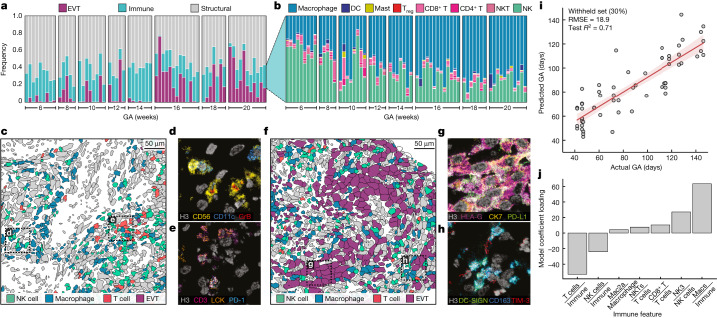
A lymphoid-to-myeloid shift in immune-compartment composition is tightly correlated with GA. **a**, Frequency of EVT, immune and structural cell populations per individual, with individuals ordered by GA. GA in days is binned to weeks for visualization. **b**, Frequency of immune cell populations per individual by GA. NK, total NK cells. **c**, Representative cell phenotype map of immune composition in decidual tissue in an early (6 weeks GA) sample. **d**, Inset of **c** showing a MIBI colour overlay of NK cells with GrB expression. Representative image of *n* = 202 FOVs. **e**, Inset of **c** showing a MIBI colour overlay of T cells with PD-1 and LCK expression. Representative image of n = 145 FOVs. **f**, Representative cell phenotype map of immune composition in decidual tissue in a late (16 weeks GA) sample. Grey, other cell types. **g**, Inset of **f** showing a MIBI colour overlay of EVTs (EVT1a and EVT1b) with PD-L1 expression. Representative image of *n* = 131 FOVs. **h**, Inset of **f** showing a MIBI colour overlay of macrophages with TIM-3 expression. Representative image of *n* = 202 FOVs. **i**, Predicted versus actual GA in days for a ridge regression model trained on GA-associated immune features for a withheld test set (30%). Line, best fit; shaded region, one standard deviation. RMSE, root mean square error. **j**, Ridge regression model coefficient loadings for GA-associated immune features. Source Data

**Extended Data Fig. 6:**
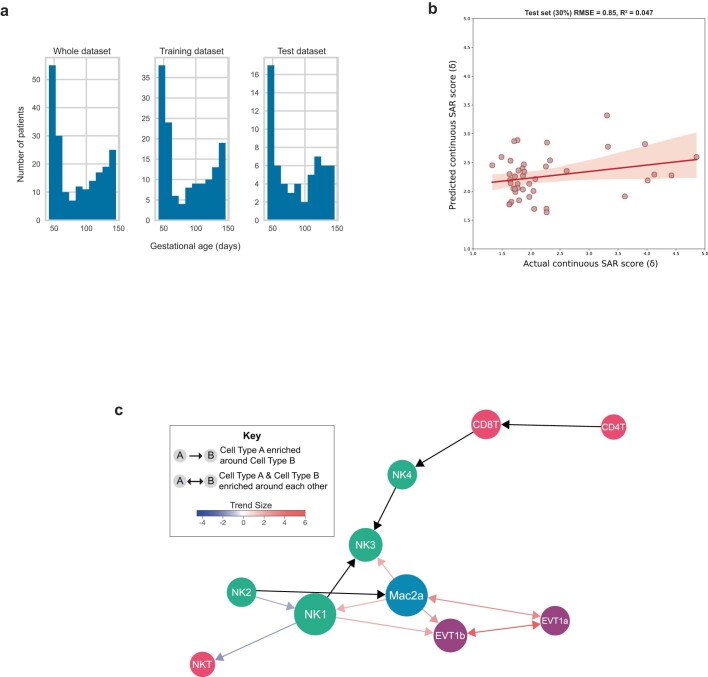
Ridge regression for predicting GA. **a**. Distribution of GA (in days) across the whole dataset, training (70%) set, and test (30%) set. **b**. Predicted versus actual δ for ridge regression trained on GA-associated immune features, for the withheld test set (30%). Line; best fit, shaded region; 1 SD. **c**. Pairwise spatial enrichment relationships, including: temporally coordinated with GA and GA/SAR synchronized. Trend size color coded: red indicates increasing spatial pairwise enrichment, blue indicates decreasing enrichment. Arrow length represents mean spatial enrichment (Z-score) across the entire dataset. Node size represents its number of pairwise relationships. Source Data

We next interrogated these data to identify GA-dependent, temporal changes in decidual composition. This analysis revealed a substantial shift from a lymphoid-dominant to myeloid-dominant landscape. Images at weeks 6–8 (Fig. 4c,e) showed NK cells and T cells exhibiting cytotoxic (Fig. 4d) and immunosuppressive (Fig. 4e) phenotypes and greatly outnumbering macrophages (Fig. 4b,c). By contrast, images from weeks 16 to 20 were dominated by interstitial EVTs (Fig. 4a,f,g) and tolerogenic macrophages (Fig. 4h). To further evaluate this relationship, we asked whether immune cell composition in the decidua alone could be used to predict GA. Using immune features that were preferentially associated with GA (Fig. 3n), we trained and validated a ridge regression model on a per-image basis using a random 70/30 test–train split (Extended Data Fig. 6a). Notably, the model predicted GA in the withheld test set within 19 days of the true value (*R*^2^ = 0.7; Fig. 4i). On inspecting the model weights, the relative contribution of decidual immune cells was consistent with the observed shift in the proportion of myeloid and lymphoid cells. Relative frequencies of T cells and NK cells were negatively correlated with GA, whereas total macrophage frequency was positively correlated with GA (Fig. 4j). Notably, a regression model for predicting SAR (*δ*) based on the same immune cell frequencies performed poorly (*R*^2^ = 0.05; Extended Data Fig. 6b), which reinforced our hypothesis that these changes are driven by GA and not SAR.

Using computational approaches validated in previous work to identify significant spatial enrichment of two cell types^22–24^ (Methods), we observed that the majority of significant pairwise enrichments involved EVT, NK cells and macrophages (Extended Data Fig. 6c and Supplementary Table 3). Again, by examining these relationships on a per-image basis, we were able to distinguish spatial relationships that evolved dynamically with respect to GA (Methods). Of these relationships, the pregnancy-specific Mac2a population was involved in the largest number of pairwise enrichments, becoming more enriched around several NK cell and EVT subsets, even though NK cells were in decline.

## Upregulation of tolerogenic markers with GA

**Fig. 5:**
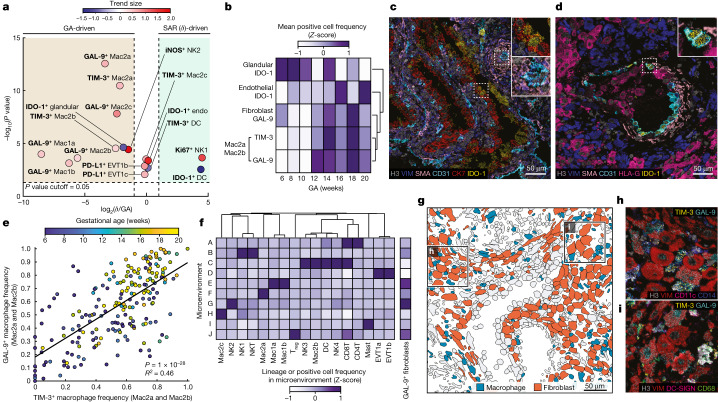
Coordinated upregulation of tolerogenic functional markers with GA. **a**, Volcano plot distinguishing GA-driven from SAR (*δ*)-driven cell-type-specific functional marker positivity fraction. *x* axis, log_2_ ratio of trend size is a relative measurement of *R*^2^ derived from linear regression against GA or SAR (*δ*) and GA. *y* axis, –log_10_ of the *P* value for the better-fitting regression model. Points are colour coded by the trend size observed in the better-fitting regression model. **b**, Heatmap of changes in a subset of GA-driven functional markers as a function of GA in weeks. GA in days is binned to weeks for visualization. **c**, MIBI colour overlay of IDO-1 expression in glandular cells (top inset) and endothelial cells (bottom inset) in an early (6 weeks GA) sample. Representative image of *n* = 122 FOVs. **d**, MIBI colour overlay of IDO-1 expression in endothelial cells (inset) in spiral artery (SAR manually assessed stage 4) of a late (16 weeks GA) sample. Representative image of *n* = 106 FOVs. **e**, Per-image Mac2a and Mac2b TIM-3^+^ cell frequency versus Mac2a and Mac2b GAL-9^+^ frequency coloured by GA. **f**, Lineage composition of cellular microenvironments across the cohort, and frequency of GAL-9^+^ fibroblasts in each microenvironment. **g**, Cell phenotype map of macrophages and decidual fibroblasts. **h**, Inset of **g**. MIBI colour overlay of TIM-3^+^ and GAL-9^+^ Mac2b cells and fibroblasts. Representative image of *n* = 153 FOVs. **i**, Inset of **g**. MIBI colour overlay of TIM-3^+^ and GAL-9^+^ Mac1a, Mac1b and Mac2a cells and fibroblasts. Representative image of *n* = 148 FOVs. Source Data

Having examined the influence of GA and SAR in driving changes in the frequency of cell populations in the decidua, we next used a similar approach to understand how these two time axes correlate with shifts in functional marker expression. These data revealed three overarching trends. First, both SAR and GA were associated with dynamic changes in IDO-1 expression. We identified a GA-driven decline in IDO-1^+^ glandular cells, in line with previous observations of IDO-1^+^ glandular cells in the first trimester but not at term^25^. We also observed a SAR-driven decline in IDO-1^+^ dendritic cells and an increase in IDO-1^+^ vascular endothelium that was comparably correlated with both GA and SAR (Fig. 5b,d). Second, consistent with the cell frequency analysis (Fig. 3n) in which NK1 cells exhibited a preferential increase with SAR, NK1 cells also exhibited a similar increase in Ki67^+^ frequency, becoming more proliferative as SAR progressed (Fig. 5a). Third, functional shifts in innate immunity were preferentially correlated with GA. All five macrophage populations upregulated either TIM-3 and/or its cognate ligand galectin-9 (GAL-9) with GA (Fig. 5a,b). This trend was most prominent in the Mac2a and Mac2b populations, in which a tightly correlated upregulation of both TIM-3 and GAL-9 was observed (Fig. 5e,g,h and Supplementary Information).

Notably, GAL-9 upregulation was also detected in fibroblasts at 12–20 weeks GA (Fig. 5b,g–i). In previous work, interactions between maternal immune and stromal cell populations have been implicated in the promotion of fetal tolerance^26^. With this in mind, we next sought to determine whether the GAL-9^+^ fibroblasts subset was biased to colocalize within specific spatial niches. To answer this question, we quantified their frequency within ten tissue microenvironments that were identified by clustering the cell-type compositions of the closest neighbours of each cell (Methods). GAL-9^+^ fibroblasts were strongly biased to colocalize with CD57^+^ NK cells (NK2, microenvironment G; Fig. 5f). Notably, this trend was accompanied by a GA-dependent increase in the expression of inducible nitric oxide synthase (iNOS) in NK2 cells (Fig. 5a). Both TIM-3 and GAL-9 have been implicated in the suppression of antitumour surveillance by impairing the activity of cytotoxic NK cells and T cells in various human cancers^27^. Together with the transient perivascular enrichment of NK2 cells observed in early SAR, these findings suggest that expression of these proteins by macrophages and fibroblasts could have a concerted tolerizing role with fetal EVTs to attenuate immune cytotoxicity subsequent to NK-cell-dependent disruption of arterial smooth muscle.

## Spatiotemporal EVT phenotype

**Extended Data Fig. 7:**
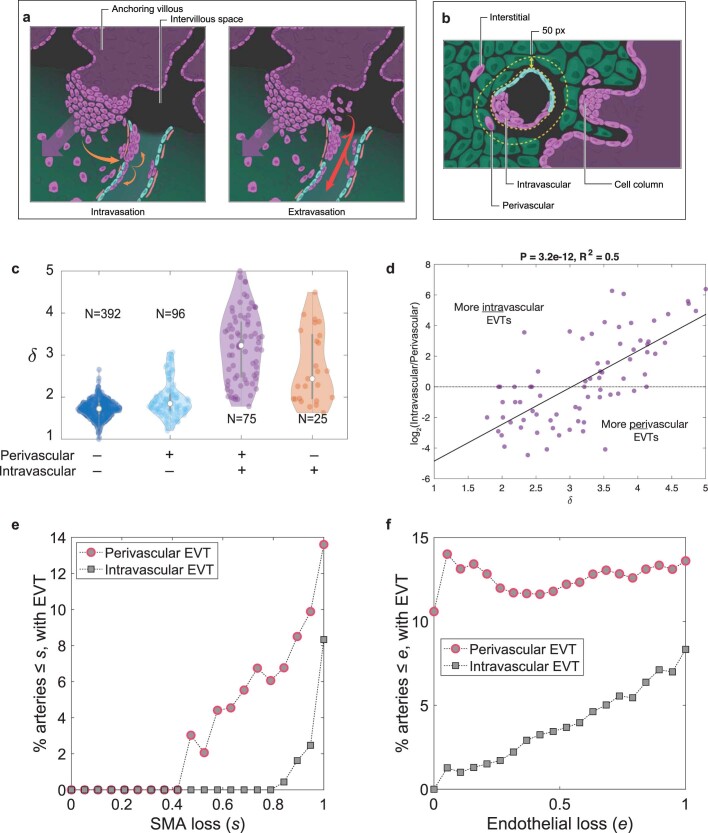
EVT distribution. **a**. Two hypotheses for intravascular EVT invasion. (Left) Intravasation: orange arrows indicate movements of EVTs from the cell column of the anchoring villi into the decidua and through the wall of the artery into the lumen. (Right) Extravasation: red arrows indicate movement of EVTs from the fetal villi through the intervillous space into the artery. **b**. Anatomical locations of EVT populations in the decidua. **c**. Distribution of artery δs, based on the presence of perivascular and/or intravascular EVTs. [Violins left to right] min = 1, max = 2.65, center = 1.7; min = 1.19, max = 3, center = 1.84; min = 1.77, max = 5, center = 3.2; min = 1.6, max = 4.5, center = 2.4. **d**. Scatter plot of log_2_(Intravascular/Perivascular) ratio by δ, for arteries with both perivascular and intravascular EVTs present. Black line, fitted linear regression. Regression p-value on top. **e**. Percentage of arteries with ≤ a given SMA loss (s) threshold, by perivascular or intravascular EVTs present. Arteries were considered to have perivascular or intravascular EVT if the number of EVT in the appropriate compartment was ≥ 5. **f**. Percentage of arteries with scores ≤ a given endothelial loss (e) threshold, by perivascular or intravascular EVTs present. Arteries were considered to have perivascular or intravascular EVT if the number of EVT in the appropriate artery compartment was ≥ 5. Source Data

Although it is accepted that EVT plugs formed during early gestation near the intervillous space are derived from the cell columns, it is unclear whether intravascular EVTs found deeper in the decidua basalis share this origin. These cells could also arrive at this location through intravasation—a migratory route by which interstitial EVTs within the decidua invade the vascular lumen by transiting across the arterial wall^3^ (Extended Data Fig. 7a).

**Fig. 6:**
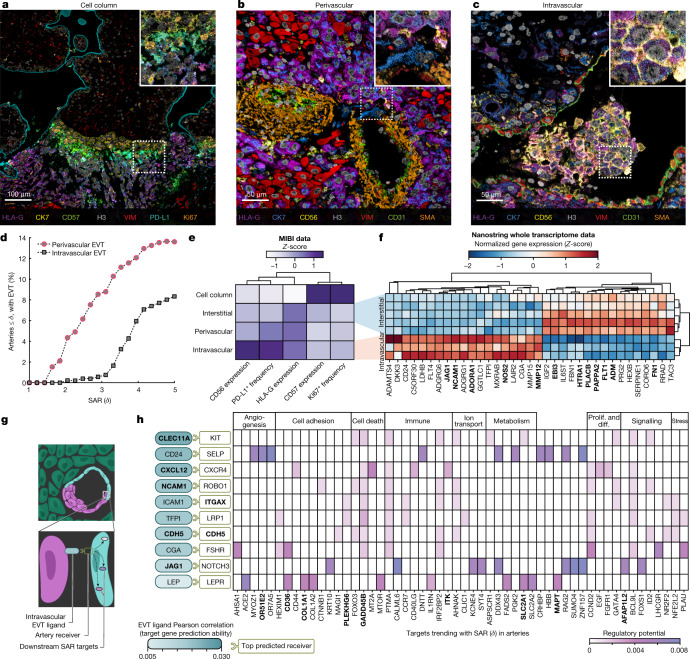
Spatiotemporal EVT distributions suggest that intravasation is the predominant route of EVT invasion in superficial decidua. **a**, MIBI overlay of anchoring villous and associated cell column EVT populations. Inset, cell column EVTs. Representative image of *n* = 60 FOVs. **b**, MIBI overlay of spiral arteries and associated perivascular EVT populations. Inset, perivascular EVT breaching artery wall. Representative image of *n* = 54 FOVs. **c**, MIBI overlay of remodelled spiral arteries and associated intravascular EVT populations. Inset, intravascular EVTs in a clump. Representative image of *n* = 23 FOVs. **d**, Percentage of arteries with scores less than or equal to a given SAR (*δ*) threshold by perivascular or intravascular EVTs present. Arteries were considered to have perivascular or intravascular EVT if ≥ 5 EVTs were present. **e**, Lineage and functional marker trends of EVT populations by anatomical location using MIBI data. Lineage marker (CD57, HLA-G and CD56) trends are mean expression values. Functional marker (Ki67 and PD-L1) trends are mean positive cell frequencies. Columns *Z*-scored and hierarchically clustered. **f**, Expression (*Z*-score) of top 35 DEGs by log(fold change) (adjusted *P* value < 0.05) between interstitial and intravascular EVT populations using NanoString whole transcriptome data. Genes also differentially expressed in preeclamptic decidua samples^1,43^ are indicated in bold. **g**, Application of NicheNet algorithm to artery and intravascular EVT whole transcriptome data to predict EVT–artery interactions and downstream signalling targets. **h**, Outcome of ligand activity prediction according to NicheNet on DEGs on intravascular EVTs. Results are shown for the ten EVT ligands that best predict receivers expressed in arteries, ranked by Pearson’s correlation coefficient or the EVT ligand activity ranking metric. Ligands, receivers and targets also differentially expressed in preeclamptic decidua samples1 are indicated in bold. Prolif. and diff., proliferation and differentiation. Source Data

To examine this possibility, we used our spatiotemporal atlas to quantify how the phenotype and spatial distribution of EVTs evolve with respect to SAR. First, we manually defined feature masks in our images to demarcate cell columns and three decidual compartments: interstitial, perivascular and intravascular (Fig. 6a–c and Extended Data Fig. 7b). We then quantified EVT frequency in each. Together with our SAR temporal trajectory, we first used these data to answer a question that has been qualitatively explored in previous work^28^. That is, whether the initial accumulation of EVTs is in the perivascular compartment (adjacent to arteries) or within the intravascular compartment. Perivascular EVTs were consistently present earlier in SAR than intravascular EVTs (Fig. 6d). Furthermore, out of all arteries with intravascular EVTs, 75% also had perivascular EVTs, which is a higher percentage than would be expected if retrograde migration was the primary source of intravascular EVTs (Extended Data Fig. 7c). In arteries where both types of EVTs were present, the ratio of intravascular to perivascular EVTs followed a smooth trend with respect to SAR, such that intravascular EVTs increased at the expense of perivascular EVTs (*R*^2^ = 0.5, *P* = 9 × 10^−12^; Extended Data Fig. 7d).

Loss of smooth muscle and endothelium have defining roles in determining the extent of SAR. Using morphometrics to quantify these cell types, we examined how the integrity of these concentric layers relates to EVT enrichment (Methods). Perivascular EVTs were consistently present at an earlier stage, whereas intravascular EVTs appeared only after 80% of smooth muscle was lost (Extended Data Fig. 7e). Notably, intravascular EVTs increased proportionally with endothelial loss, whereas perivascular EVTs were present irrespective of this process (Extended Data Fig. 7f). Together, these findings align with a process whereby near-complete loss of smooth muscle permits perivascular EVTs to invade the artery lumen through discontinuities in the vascular endothelium^29^.

**Extended Data Fig. 8:**
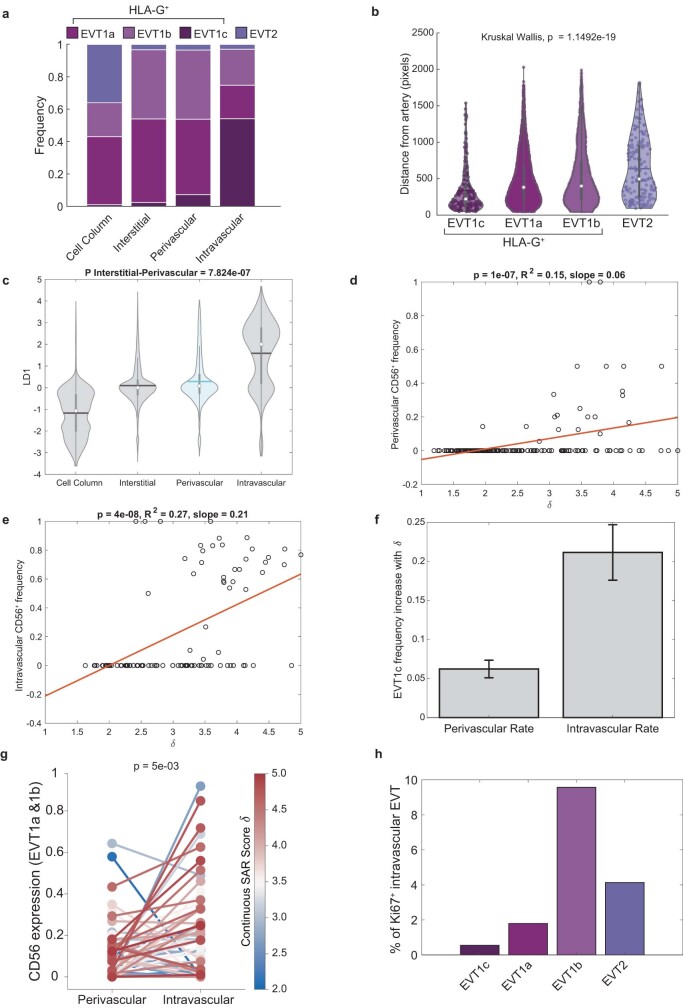
EVT Phenotype by Location. **a**. Frequency of EVT populations by anatomical location. **b**. Violin plot of distance from artery (in pixels) by EVT type. EVT1c: N = 209, min = 12.8, max = 1536.7, center (median) = 173.9; EVT1a: N = 7123, min = 10, max = 2028.7, center = 363.6; EVT1b: N = 5908, min = 7.2, max = 1989.5, center = 376.45; EVT1c: N = 185, min= 9.4, max. = 1818.1, center = 475.6. **c**. Violin plot of the distribution of LD1 for EVTs, by anatomical location. Center lines inside violins indicate mean. Anchoring: N = 8906, min = −3.6, max = 3.9, center = −1; Interstitial: N = 38395, min = −3.4, max = 4.4, center = 0; Perivascular: N = 1097, min = −3.14, max = 4, center = 0.08; Intravascular: N=4040, min = −3.15, max = 4.6, center = 2. **d**-**h**: **d**,**e** Scatter plots of perivascular (**d**) and intravascular (**e**) EVT1c (CD56+) frequency by δ. Red lines, fitted linear regression, regression p-values on top. **f**. Bar plot of the EVT1c frequency increase rates (regression slopes from **d**,**e**). Error bars, 95% confidence interval for regression slopes. **g**. Paired-by-artery CD56 expression in EVT1a&b, between perivascular and intravascular compartments. Arteries with δ ≥ 2 included. p = 5e-03, one sided paired Wilcoxon signed rank test, z-statistic = 2.5 h. Proportion of Ki67+ intravascular EVT by type. Source Data

To further evaluate this model, we next examined whether the EVT phenotype shifts progressively in a manner consistent with a migratory route that passes through the decidua into the artery lumen. The proportion of EVT subsets in cell column, interstitial, perivascular and intravascular compartments shifted systematically in a manner consistent with this proposed path of migration (Extended Data Fig. 8a,b). Examining functional marker expression within each compartment again revealed a progressive shift in EVT phenotype. Cell columns were distinctly enriched for proliferative (Ki67^+^) and CD57^+^ EVTs. A progressive decrease in these markers across interstitial, perivascular and intravascular EVTs was accompanied by a concomitant increase in PD-L1 that peaked in the intravascular compartment (Fig. 6e, Extended Data Fig. 8c and Methods).

These analyses collectively align with a spatial trajectory whereby decidual invasion of cell column EVTs is synchronized with a downregulation of CD57 and Ki67 and an upregulation of HLA-G. Perivascular accumulation of EVTs occurs early in SAR before the appearance of intravascular EVTs and any loss in endothelium. In this model, as the endothelial barrier is lost, perivascular EVTs invading the artery lumen upregulate CD56 and PD-L1 (Extended Data Fig. 8d–h and Methods). Notably, owing to its observational nature, this study cannot rule out alternative mechanisms by which detaching EVTs arising from EVT plugs at the intervillous space undergo retrograde migration.

**Extended Data Fig. 9:**
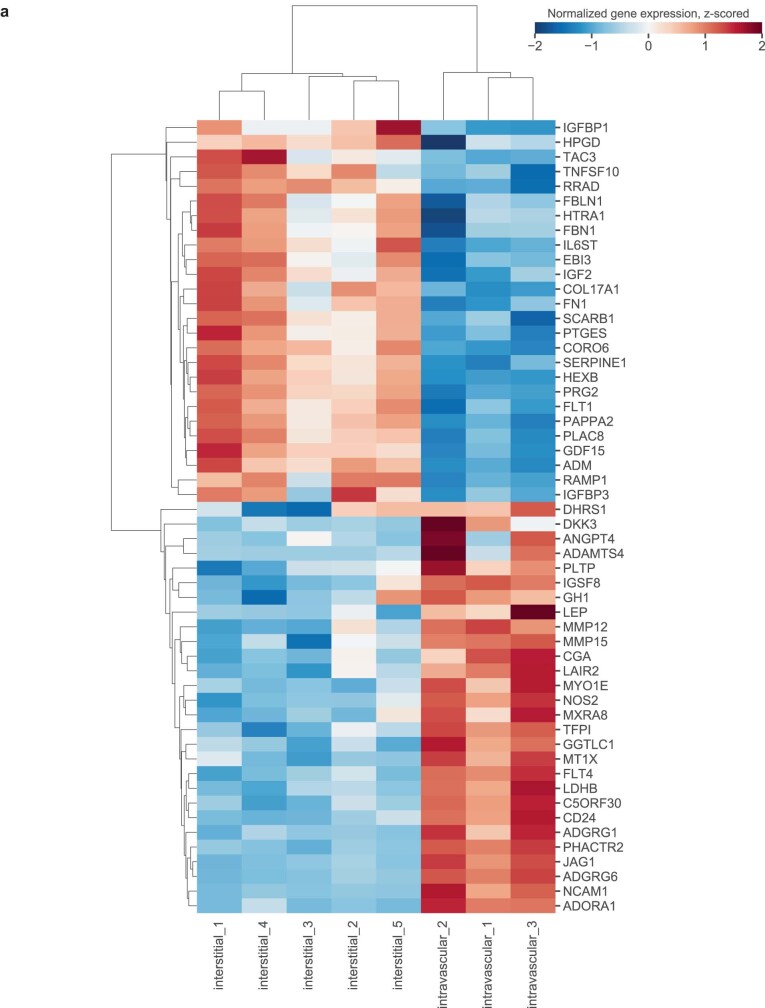
Differential gene in EVT. **a**. Full heatmap for differentially expressed genes between intravascular and interstitial EVTs showing gene expression (Z-score), (logFC > 2, adj p-value <0.05 using Limma). Source Data

**Extended Data Fig. 10:**
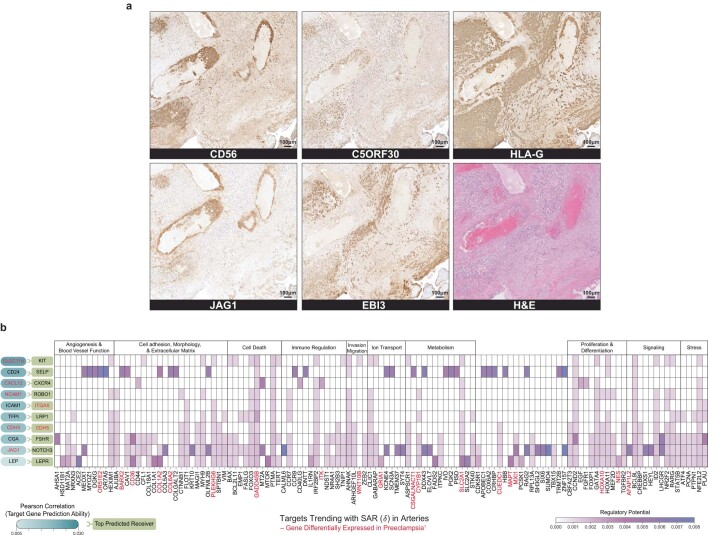
EVT- artery interactions. **a**. IHC validation on decidua serial sections for protein counterparts for 3 differentially expressed genes (JAG1, C5ORF30, and EBI3) with controls: HLA-G, CD56, and H&E. 1 section per marker. Scale bar, 100 µm. **b**. Full NicheNet output: 10 EVT-ligands best predicting receivers expressed in arteries, ranked by Pearson correlation coefficient or the EVT ligand activity ranking metric. Genes differentially expressed in Preeclamptic decidua samples indicated in red. Source Data

Irrespective of the migration route, the distinct changes in phenotypic markers measured by MIBI-TOF suggest that arterial invasion is accompanied by a shift in EVT transcriptional programmes. With this in mind, we used NanoString DSP to measure the transcriptomes of interstitial and intravascular EVTs. We found 274 differentially expressed genes (DEGs) (Extended Data Figs. 9 and 10a and Methods). In addition to confirming previous work noting an upregulation of *NCAM1*, *JAG1* and *LAIR2* specifically in intravascular EVTs^30–32^, we identified transcriptional changes in genes important for extracellular matrix remodelling and angiogenesis (Fig. 6f). For example, *MMP12*, *MMP15* and *ADAMTS4* were specifically upregulated in intravascular EVTs (mean log(fold change) of 11.67 for *MMP12*, 9.74 for *MMP15*, and 7.88 for *ADAMTS4*), which suggested that these proteins play a significant role in late-stage SAR. In addition, arterial invasion was accompanied by a shift from *VEGFR1* (also known as FLT1) to *VEGFR3* (also known as FLT4) (Fig. 6f).

Intravascular EVTs also upregulated *DKK3*, *C5orf30* (also known as MACIR) and *CD24* (Fig. 6f), which have each been shown in previous work to have roles in fetal viability, tumour invasion or immune tolerance^33–35^. Similarly, we observed an accompanying downregulation of genes associated with invasion in intravascular EVTs, such as *MGAT5*. With respect to immune modulation, C5orf30 is a potent immunometabolic regulator that has been shown to inhibit macrophage-mediated tissue damage in rheumatoid arthritis^33^. Similarly, CD24 binding to Siglec-10 was recently found in many cancers to promote immune evasion by serving as an anti-phagocytic, ‘don’t eat me’ signal^36^. Taken together, our multimodal approach paints a picture of a highly regulated and controlled process. We observe a transcriptional shift away from a more invasive phenotype (*SERPINE1* and *CORO6*) in interstitial EVTs towards genes implicated in vascular remodelling in intravascular EVTs. Notably, this was accompanied by an increase in immunoregulatory modules that allow EVTs to be in continuous contact with maternal blood while avoiding immune activation (Fig. 6e,f).

To understand how these changes promote SAR, we investigated potential cell–cell interactions between intravascular EVTs and arterial cells using NicheNet^37^ (Methods). We identified ten protein–protein interactions between EVTs and arteries that were predicted to affect 121 downstream targets (Fig. 6g,h, Extended Data Fig. 10b and Supplementary Table 5). For example, interactions between EVT JAG1 and arterial Notch were predicted to drive downstream changes in arterial MEOX1 and MT2A, which have been implicated in endothelial dysfunction and apoptosis^38,39^. Similarly, CGA–FSHR and LEP–LEPR interactions correlated with changes in arterial hormone receptors (LHCGR) and several cell adhesion targets, respectively (Fig. 6h and Extended Data Fig. 10b). Notably, among the most prominent downstream targets were the olfactory receptors OR51E2 and the human-specific OR7A5, the expression of which outside the olfactory bulb has been thought to regulate blood pressure and angiogenesis^40,41^.

CD24–SELP was the second most significant interaction and had several targets related to blood vessel function and formation (Fig. 6h and Extended Data Fig. 10b). Notably, reduced *CD24* mRNA levels in bulk placental samples has been associated with higher preterm preeclampsia risk^35^; however, EVT-specific CD24 expression has not been previously reported. Given that abnormal decidual and SAR are thought to play a major part in preeclampsia^42^, we sought to determine whether other genes involved in EVT invasion and vascular remodelling had previously been implicated. To do this, we first compared our list of EVT DEGs with genes found previously to be differentially expressed in decidua samples from women diagnosed with preeclampsia^43^. We found that 31% of EVT DEGs (12 genes) are differentially expressed in preeclamptic decidua (Fig. 6f and Supplementary Table 6). Notably, *FN1* and *FLT1*, which have been proposed as biomarkers for early prediction of preeclampsia^44,45^, were markedly downregulated in intravascular EVTs.

Half of the NichNet interactions and 19 downstream targets overlapped with this list of preeclampsia DEGs^43^ (Fig. 6h and Extended Data Fig. 10b). These included *WNT10B*, a newly identified accelerator of EVT invasion^46^, and *OR51E2*, a target of CD24–SELP signalling that also exhibited the highest regulatory potential. With respect to the latter, SELP is notable for being differentially expressed in peripheral blood-cell-free RNA from patients with preeclampsia^47^. Taken together, our transcriptomics approach validated and complemented the stepwise changes in EVT phenotype seen in our spatial atlas while revealing pathways that are perturbed in pregnancy-related disorders.

## Discussion

Decidualization is a fascinating process with no other normative precedent in human biology. In this process, the structure and function of the maternal endometrium transforms to promote the regulated invasion of genetically dissimilar fetal cells. The decidua plays a dual role by permitting EVT invasion in the first trimester and later limiting it by inducing EVT apoptosis^48^. EVT invasion can also be limited by morphological changes such as EVT fusion, which leads to polyploidization that limits invasion owing to nuclear size^49^. Given the lack of tractable and relevant animal models and the inability to study decidualization prospectively, our understanding of it is immature relative to other areas of human physiology. Therefore, our study aimed to understand how global, temporally dependent changes in decidual composition are coupled to local regulation of vascular remodelling in pregnancy. Initial invasion of placental EVTs is prompted by a shift towards a permissive milieu, whereas progression of SAR depends on the subsequent migration and perivascular accumulation of EVTs, where they are thought to participate in cooperative cell–cell interactions with maternal fibroblasts, NK cells and macrophages^2^. Thus, the formation of the maternal–fetal interface is mediated by global, temporally dependent cues that serve as a gating function for remodelling processes that are regulated in the local tissue microenvironment.

With this paradigm in mind, we set out to delineate which aspects of the first half of pregnancy are driven globally by GA and how this relates to SAR. In the study of placentation and SAR, an ideal sampling strategy might use elective caesarean hysterectomies from normal pregnancies performed across GA in an ethnically diverse patient population. As ethical considerations prohibit this approach, previous work has used a range of sample types that each have their own strengths and weaknesses. Here we utilized archival tissue from elective terminations with no known pregnancy complications. This enabled us to examine these questions in a large, ethnically diverse cohort that is well-distributed with respect to GA. As tissue procured during terminations is fragmented, anatomical registration for determining whether these tissue blocks were sampled from central or peripheral regions of the decidua basalis was not feasible.

Using LDA, image morphometrics and expert annotations, we assigned quantitative remodelling scores to every spiral artery in these images. These targeted multiplexed imaging data were complemented by spatially co-registered tissue transcriptomics. This multimodal dataset enabled us to reveal how cell frequency and function, tissue organization and transcriptional programmes in maternal decidua, arteries and EVTs change with SAR and GA.

Our analyses of these changes determined that GA is the predominant driver of maternal immune cell recruitment. Progressive decreases in the numbers of NK cells and T cells drive a transition at 12–14 weeks GA from a lymphoid-dominant to myeloid-dominant decidua enriched for iNOS^+^ NK cells, IDO-1^+^ vascular endothelium and DC-SIGN^+^ macrophages that express both TIM-3 and GAL-9. Notably, this relationship between immune composition and GA was strong enough to allow us to predict GA within 19 days exclusively on the basis of immune population frequencies.

By contrast, all EVT subsets and only two maternal cell populations (NK1 and NK2) preferentially correlated with SAR. Higher remodelling scores were correlated with more EVTs, more NK1 cells and fewer NK2 cells. A sharp accumulation in NK2 cells around arteries was observed early in the remodelling process around the time smooth muscle disruption had been initiated. NK1 and NK2 cells primarily differed in that the latter express CD57, a marker associated with a cytotoxic phenotype. Higher proportions of presumptively more reactive NK2 cells early in SAR around arteries aligns well with previous results^50^ that have suggested that decidual NKs initiate early disruption of arterial smooth muscle through the secretion of GrB, MMP2 and MMP9. Likewise, the proportional gains seen here as SAR progresses of less reactive NK1 cells and invasive EVTs are consistent with the tolerizing effects of HLA-G, which has previously been shown^51,52^ to decrease NK cell cytotoxicity and induce the production of interleukin-6 and interleukin-8 through the binding of HLA-G to KIR2DL4, LILRB1 and LILRB2. Taken together, these data suggest that maternal and fetal cells have cooperative, interdependent roles with SAR, transitioning through NK-dependent and EVT-dependent phases.

We also examined a lingering question^3^ in the field: the path of migration taken by intravascular EVTs in the decidua basalis. In line with early work based on studies of 8–18 week hysterectomy specimens processed in toto^3^, we found that perivascular EVTs accumulated before intravascular EVTs. By comparing the cellular composition within cytotrophoblast cell columns of anchoring villi, decidua and arteries, we observed a sequential and coordinated shift in EVT frequency and phenotype, which provided support for a model in which EVTs enter spiral arteries from nearby decidua by crossing the arterial wall.

Notably, previous studies of samples from caesarean hysterectomies identified morphological evidence of arterial extravasation^3^. Given the observational nature of this study and the limited preservation of tissue structure at the intravillous space, an extravasation model in which EVTs migrate in a retrograde manner after entering spiral arteries directly at the basal plate cannot be excluded. We also note the possibility that following intravasating arteries in the decidua, EVTs could migrate upstream to reach the upper third of the myometrium. This idea would be consistent with previous studies in which perivascular trophoblasts become increasingly scarce as a function of myometrial depth^53^. Therefore, it is conceivable that both processes may be at play in different regions of the decidua as pregnancy progresses.

Previous single-cell and bulk sequencing studies of decidua have characterized the transcriptome of decidual cells; however, they were performed using dissociated tissue, agnostic to spatial context and the local extent of SAR^2,12^. Correlating spatial morphology and tissue composition with targeted tissue transcriptomics enabled us to observe how the transcriptome evolves with respect to SAR. In arteries, our analysis revealed a downregulation of Notch signalling, tissue organization and cohesion with SAR, which was accompanied by a burst of translation-related activity around stage 2 of remodelling. By comparing interstitial EVTs with intravascular EVTs, our analyses revealed genes upregulated in the interstitial populations that shed light on how EVTs facilitate immune tolerance. Almost one-third of DEGs between interstitial and intravascular EVTs overlapped with DEGs in preeclamptic decidua samples. Given the significant contribution that abnormal vascular remodelling and EVT invasion are thought to play in preeclampsia, this work serves as a valuable resource for contextualizing preeclampsia-related changes in future studies.

Notably, many of these pathways are also associated with cancer progression. Formation of the maternal–fetal interface is an organized and controlled invasive process that is sometimes viewed as a template for understanding invasive and immunosuppressive properties of tumours^54^. Both processes involve a genetically dissimilar invasive cell type (haploidentical EVTs versus clonal, mutated cancer cells), extracellular matrix remodelling and recruitment of a wide variety of tolerogenic immune cells, including M2-polarized macrophages and proliferating T_reg_ cells. The intersection of anchoring placental villi and maternal decidua morphologically resembles the invasive margin of carcinomas and contains trophoblast cells that express high levels of immunomodulatory proteins and growth factors implicated in tumour severity, including PD-L1, IDO-1, TIM3, HER2 and EGFR^27,55,56^. In addition to these phenotypic and structural similarities, recent work that revealed mosaicism and clonal mutations in normal-term placentas demonstrated that this phenotypic overlap is even manifest at a genomic level^57^.

Overall, we anticipate that this spatiotemporal atlas of the early human maternal–fetal interface will provide a normative framework for elucidating aetiological perturbations in maternal–fetal tolerance and SAR in pregnancy complications. Likewise, this work may also serve as a template for understanding how immune tolerance, tissue remodelling and angiogenesis are aberrantly recruited and synergized during tumour progression. With this in mind, we plan in future studies to extend this comparative approach to archival tissue in the context of obstetric complications to further elucidate cellular interactions involved in the regulation of SAR and EVT invasion.

## Methods

### Retrospective cohort design

The study cohort comprised decidua tissue from archival formalin-fixed, paraffin embedded (FFPE) blocks, sampled after elective pregnancy terminations from an outpatient clinic located within a large public hospital affiliated with an academic medical centre. Patients at this clinic reflect a diverse population. Although the patient population is predominantly low-income, women of all economic backgrounds are cared for at the clinic.

In the clinic, an ultrasound examination is performed to estimate GA, and a medical history is taken and logged as an electronic medical record (electronic clinical works) or handwritten forms. A board-certified gynaecologist reviewed medical records and specifically extracted the following details: age, ethnicity, body–mass index, gravidity, parity, previous terminations, smoking status, medications, HIV status, history of preeclampsia, chronic hypertension, diabetes mellitus, renal disease, autoimmune disease, multifetal pregnancy, and congenital anomalies (Supplementary Table 1). For procedures occurring at <14 weeks GA, suction aspiration is routinely used. For procedures at >14 weeks GA, a combination of suction aspiration and grasping forceps is used. After the procedure, tissue samples are routinely sent to pathology.

### TMA construction

Whole tissue sections from individuals who underwent elective termination at 6–20 weeks of gestation were first reviewed by H&E staining to identify samples containing decidual tissue and spiral arteries. These regions were manually demarcated and assessed for suitability. Blocks containing decidua with vessels were selected, cored with a bore needle and assembled into the TMA used in this study. Archival tissue blocks from 74 individuals were initially selected by a board-certified perinatal pathologist (G.R.) to be included in the TMAs. The first TMA consisted of 205 cores (including 3 tonsil cores, 1 endometrium core and 1 myometrium core) of 1 mm in diameter and the second contained 86 cores of 1.5 mm in diameter). Unfortunately, cores from eight individuals did not end up containing decidua, and there was not sufficient tissue in the block for additional re-coring. We therefore had to exclude these samples from the analysis. The final cohort included 66 individuals, an exhaustive list of which is provided in Supplementary Table 1. Images from samples from six individuals did not have arteries and therefore were not included in analyses related to spiral arteries. Information on the histological characteristics of the blocks retrieved, including the presence of cell column anchoring villi, is in Supplementary Table 1. High-resolution scans of each core were uploaded to the Stanford Tissue Microarray Database (http://tma.im/cgi-bin/home.pl), a collaborative internal platform for designing, viewing, scoring and analysing TMAs. Sequential recuts of the main experiment were stained with H&E to aid in choosing the imaging ROIs and analysing data.

### Antibody preparation

Antibody staining was validated as previously described^11,58^. In brief, each reagent was first tested using single-plex chromogenic immunohistochemistry (IHC) using multiple positive and negative FFPE tissue controls before metal conjugation. Antibodies were then conjugated to isotopic metal reporters as previously described^11,22–24,58^ with the exception of biotin-conjugated anti-PD-L1, for which a metal-conjugated secondary antibody was used. The performance of metal-conjugated antibody reagents were then tested within the complete MIBI-TOF staining panel under conditions identical to those in the main study and compared with representative single-plex chromogenic IHC to confirm equivalent performance. Representative stains and information for each marker is provided in the Supplementary Information and in Supplementary Table 7, respectively. After conjugation, antibodies were diluted in Candor PBS Antibody Stabilization solution (Candor Bioscience). Antibodies were either stored at 4 °C or lyophilized in 100 mM d-(+)-trehalose dehydrate (Sigma Aldrich) with ultrapure distilled H_2_O for storage at −20 °C. Before staining, lyophilized antibodies were reconstituted in a buffer of Tris (Thermo Fisher Scientific), sodium azide (Sigma Aldrich), ultrapure water (Thermo Fisher Scientific) and antibody stabilizer (Candor Bioscience) to a concentration of 0.05 mg ml^–1^. Information on the antibodies, metal reporters and staining concentrations is in Supplementary Table 7.

### Tissue staining

Tissues were sectioned (4 μm in thickness) from tissue blocks on gold and tantalum-sputtered microscope slides. Slides were baked at 70 °C for 20 min followed by deparaffinization and rehydration with washes in xylene (3 times), 100% ethanol (2 times), 95% ethanol (2 times), 80% ethanol (once), 70% ethanol (once) and ddH_2_O with a Leica ST4020 Linear Stainer (Leica Biosystems). Tissues next underwent antigen retrieval, which was carried out by submerging sides in 3-in-1 Target Retrieval solution (pH 9, Dako Agilent) and incubating them at 97 °C for 40 min in a Lab Vision PT Module (Thermo Fisher Scientific). After cooling to room temperature, slides were washed in 1× PBS IHC washer buffer with Tween 20 (Cell Marque) with 0.1% (w/v) BSA (Thermo Fisher). Next, all tissue samples underwent two rounds of blocking, the first to block endogenous biotin and avidin with an Avidin/Biotin Blocking kit (BioLegend). Tissue samples were then washed with wash buffer and blocked for 1 h at room temperature with 1× TBS IHC wash buffer with Tween 20 and 3% (v/v) normal donkey serum (Sigma-Aldrich), 0.1% (v/v) cold fish skin gelatin (Sigma Aldrich), 0.1% (v/v) Triton X-100 and 0.05% (v/v) sodium azide. The first antibody cocktail was prepared in 1× TBS IHC wash buffer with Tween 20 and 3% (v/v) normal donkey serum (Sigma-Aldrich) and filtered through a 0.1 μm centrifugal filter (Millipore) before incubation with tissue overnight at 4 °C in a humidity chamber. After overnight incubation, slides were washed for 2 min in wash buffer. The next day, the antibody cocktail was prepared as described (Supplementary Table 7) and incubated with the tissues for 1 h at 4 °C in a humidity chamber. After staining, slides were washed twice for 5 min in wash buffer and fixed in a solution of 2% glutaraldehyde (Electron Microscopy Sciences) solution in low-barium PBS for 5 min. Slides were washed in low-barium PBS for 20 s then, using a linear stainer, through 0.1 M Tris at pH 8.5 (3 times), ddH_2_O (2 times) and then dehydrated by washing in 70% ethanol (once), 80% ethanol (once), 95% ethanol (2 times) and 100% ethanol (2 times). Slides were dried under vacuum before imaging.

### MIBI-TOF imaging

Imaging was performed using a custom MIBI-TOF instrument with a Xe^+^ primary ion source, as previously described^22,58^. In total, 222 808 × 808 µm FOVs were acquired at approximately 600 nm resolution using an ion dose of 7 nA × h mm^–2^. After excluding 11 FOVs that contained necrotic or non-decidual tissue, or consisted of duplicate tissue regions, the final dataset consisted of 211 FOVs from 66 individuals.

### Low-level image processing

Multiplexed image sets were extracted, slide background-subtracted, denoised and aggregate filtered as previously described^22–24,58,59^. For several markers, a background channel consisting of signal from the mass 128 channel was used. All parameters used as inputs for low-level processing are listed in Supplementary Table 7.

### Feature annotation

Large tissue features were manually annotated in collaboration with a perinatal pathologist. Pseudo-coloured MIBI images stained with H3 to identify cell nuclei, VIM for decidual stromal cells, SMA and CD31 for vessels, cytokeratin 7 (CK7) for glands and the fetal cell columns, and HLA-G for EVTs were used to guide annotation. Serial H&E sections, and a H&E recut of the entire block, if necessary, were additionally used to supplement annotation. Labelling was performed in ImageJ and the annotated features were exported as binary TIF masks.

### Single-cell segmentation

The Mesmer segmentation algorithm^60^ was adapted specifically to segment the cells in our dataset. First, training data were generated using a subset of 15 images out of 211 in our cohort, in addition to 10 decidua MIBI-TOF images from titration data. In total, 1,024 × 1,024 pixel crops were selected to encompass the range of different cell morphologies present. The markers H3, VIM, HLA-G, CD3, CD14 and CD56 were used to capture the major cell lineages present. Subsequently, a team of annotators parsed these images to identify the location of each unique cell using DeepCell Label, custom annotation software specifically developed for this task^60^ (https://github.com/vanvalenlab/deepcell-label). The manually annotated images were used to generate partially overlapping crops of 256 × 256 pixels from each image. In total, training data included 1,600 distinct crops with 93,000 cells. This dataset was used to retrain the Mesmer segmentation model, modifying the architecture to accept six distinct channels of input. The output from the network was then post-processed using the default model settings (Supplementary Information).

### Segmentation post-processing

Examination of the images revealed that glandular cells and chorionic villus trophoblasts did not express any markers included in the training data; namely these cells were predominantly CK7^+^. This resulted in effectively nuclear-only segmentation being predicted by the convolutional neural network within these features. To account for this, segmented cells that overlapped with the gland mask were expanded radially by 5 pixels, and those in the cell column mask by 2 pixels. The number of pixels used for expansion was optimized to approximate the observed cell size, which was based on a systematic inspection of three images per GA. Objects <100 pixels in area were deemed noncellular and excluded from subsequent analyses. The final number of segmented events per FOV is provided in Supplementary Table 8.

### Single-cell phenotyping and composition

Single-cell expression data were extracted for all cell objects and area-normalized. Single-cell data were linearly scaled with a scaling factor of 100 and ArcSinh-transformed with a co-factor of 5. All mass channels were normalized to the 99th percentile. To assign decidual cell populations (≥70% cell area in decidua) to a lineage, the clustering algorithm FlowSOM (Bioconductor FlowSOM package in R)^29^ was used, which separated cells into 100 clusters based on the expression of 19 canonical lineage-defining markers (Supplementary Information). Clusters were further classified into 21 cell populations, with proper lineage assignments ensured by manual examination of overlayed FlowSOM cluster identity with lineage-specific markers. Clusters containing non-biologically meaningful or distinct signals were assigned the label ‘other’. T_reg_ cells were identified by thresholding T cells (FlowSOM clusters 43, 53 and 63) with the CD3 signal ≥ the mean CD3 expression of CD4^+^ T cells and >0.5 the normalized expression of FOXP3. Mast cells were identified as cells for which normalized expression of tryptase was >0.9. Mac2b (CD11c^+^) cells were identified as macrophages with >0.5 normalized expression of CD11c. Placental macrophages (Hofbauer cells) were defined as CD14^+^ >0.5 cells located within the cell column. Cells from FlowSOM clusters 4, 5 and 15 ubiquitously and predominantly expressed CK7 and were reassigned to the EVT2 subset if located within the cell column feature mask or as glandular cells otherwise (Supplementary Information). These thresholds were selected based on the distribution of lineage marker expression (Supplementary Information) and on systematic examination of the images by eye as expression patterns varied significantly between markers. For a comprehensive list of all single cells, their morphological features, markers expression, lineage classification, among others, see the Data availability section.

### Definition of thresholds for functional marker positivity

Cells were considered positive for a functional marker if their scaled expression level was greater than or equal to a set threshold, as previously described^22^. Thresholds for individual functional markers were determined on the basis of examining the images by eye, as expression patterns varied significantly between markers (Supplementary Table 9 and Supplementary Information). To set the per-marker thresholds, five images for each functional marker were reviewed, and increasing threshold values were examined using custom software. Subsequently, cells defined as negative for a marker based on the determined threshold value were re-examined to ensure that the thresholds were representative. For Ki67 positivity, only cells that had a nucleus in the image were considered. Ki67 values were not normalized to the cell size because the Ki67 signal is exclusive to nuclei.

### Two-colour IHC

Before staining, FFPE sections were incubated at 70 °C for 1 h. After deparaffination and antigen retrieval (Dako, S2367) was performed, endogenous horseradish peroxidase and alkaline phosphatase were blocked using BLOXALL (Vector Laboratories, SP-6000-100) for 30 min, followed by blocking buffer solution (95% 1× TBS IHC wash buffer with Tween 20, 1% Triton 10%, 1% gelatin 10%, 2% horse serum and 1% sodium azide 20 mg ml^–1^) for 1 h at room temperature. Double staining was performed using CD57 (mouse IgG) paired with CD49a (rabbit IgG). Sections were incubated at 4 °C overnight with the antibodies CD57 (clone NK/804, Abcam, ab269771; titre, 0.5 µg ml^–1^) and CD49a (clone E9K2J, CST, 15574T; titre, 1:1,500). The following day, secondary antibody (ImmPRESS Duet reagent; HRP anti-rabbit IgG and AP anti-mouse IgG; Vector Laboratories) was applied for 10 min at room temperature. Antibodies were revealed with Vector Blue AP substrate (Vector Laboratories, SK-5300) for 10 min in the dark followed by DAB HRP substrate (Vector Laboratories, SK-4105) for 40 s. For subsequent analyses and colour deconvolution, single-plex staining for CD57 and CD49a were performed on one slide each. For details on the method, buffers and solutions, refer to ref. ^61^.

The IHC slides were scanned using a NanoZoomer Digital Pathology Scanner 2.0RS (Hamamatsu) and analysed using QuPath (v.0.4.0). To score CD57^+^ NK cells (NK2) for expression of the tissue-residency marker CD49a, the two colours in the IHC slides were deconvolved with QuPath using single-plex staining as colour references. CD57^+^ NK cells were then manually annotated, in decidual regions only, by a board-certified pathologist. These cells were than manually scored for CD49a expression and counted.

### Blinded manual artery staging

Arteries were categorized into five remodelling stages based on criteria adapted from a previously proposed four-stage model^21^. These criteria were used to describe spiral arteries observed in H&E and single-channel IHC images and were adapted to suit multiplexed MIBI data (Fig. 3a, details in Extended Data Fig. 3a). In total, 600 arteries were categorized according to these criteria by a single reviewer using only crops of MIBI pseudocolour overlays (SMA, VIM, CD31, H3 and HLA-G), including only the artery (as defined by a feature mask) and any EVTs in the lumen. The reviewer was blinded to the rest of the image, serial H&E sections, GA and any clinical data. Twelve partially captured arteries were excluded from the final dataset of 588 arteries.

### Automated digitization of artery morphological features

The same format of cropped artery MIBI images that were manually scored by the reviewer were used to calculate a set of geometric parameters for several selected features. These features described the organization and structure of the vessel wall, the continuity of the endothelium and its thickness, and the presence and structure of intravascular EVTs. To capture these features, a structure of concentric circles we termed the ‘onion’ structure was defined, with the outer circle of this structure enclosing the artery and the inner circles dividing it into layers. This structure is described below using the two-dimensional cylindrical coordinate system, with the radial axis *r*, azimuthal (angular) axis *ø*, and origin of the axis at point (*x*,*y*). Point (*x*,*y*) is the user-defined artery centre. For an artery in the binary mask *M*, the following algorithm was used to create the onion structure (Extended Data Fig. 3c).First, define a circle enclosing the artery, centred at point (*x*,*y*) with radius *a* as follows: (*x*,*y*) was taken as the user-defined artery centre point; *a*, the radius is defined as the maximum distance between (*x*,*y*) and the edge of *M*, rounded up to the nearest integer multiple of *n*, such that *a* = *I* × *n* for an integer *I*. *n* is a user-defined thickness parameter for the onion layers

Second, define the inner circles comprising the onion layers by dividing the radius *a* of the outer circle into *I* equal sections of length *n*, creating layers along the radial *r* axis. The radii of the inner circles are then defined as 0,1 × *n*,2 × *n*,…(*I* – 1) × *n*.

Third, divide the onion into *k* equal sectors along the *ø* axis. *k* is a user-defined integer.

Fourth, subdivide each sector into segments. The sectors are internally divided by the circles, creating parts with four corners and four sides, with the two sides being straight (sector dividers), and the two sides being arcs (parts of circle circumferences). The arcs are replaced with secants (straight line connecting the ends of the arc), turning the segment into a trapezoid. The parameters *n* = 10 pixels and *k* = 100 were used to allow for segments large enough to contain a sufficient number of pixels to average the expression over.

Geometrical and protein morphology features were then extracted for each artery onion. For geometrical features, the following parameters were defined: (1) radius, the maximum distance between any pixel within the mask and the closest pixel on the edge of the mask; (2) perimeter, the Euclidean distance between all adjacent pixels on the edge of the artery mask; and (3) area, the total number of pixels within the artery mask.

For the protein morphology features, for the markers CD31, CK7, H3, HLA-G, SMA and VIM, the following parameters were defined. (1) Average signal: the weighted average over segments of marker expression, in which the weight of a segment corresponds to the number of pixels it contains. The weighted average was used to avoid smaller inner segments having a disproportionate effect on the average.(2) Thickness: for each sector, we calculated the distance *d* between the inner-most segment positive for the marker and the outer-most positive segment. Positivity was measured by comparing the mean signal over pixels the segment to a user-defined threshold.The mean and standard deviation of thickness were calculated as the mean and standard deviation of *d* over all sectors. (3) Radial coverage: the percentage of sectors positive for marker signal. A sector was considered positive if the mean signal over sector pixels acceded a user-defined threshold. (4) Jaggedness: this feature measures the extent jaggedness of an artery outline. To do so, first, a previously described skeletonization function^62^ is applied to the artery mask, and this function returns a ‘skeleton’ of the artery outline. This skeleton also assigns values to the outline pixels based on their distance from the core shape. Then, two different binarization thresholds are chosen: a non-branch threshold (a high value = 60 pixels, which indicates a greater topological distance) and a ‘branch’ threshold (a low value = 5 pixels, which indicates a smaller topological distance). The ratio between the total number of non-branch and branch pixels is the jaggedness.

### Calculation of continuous SAR remodelling score *δ*

A supervised dimensionality reduction technique based on LDA^63^ (https://github.com/davidrglass) was applied using the per-artery digitized morphological features and manually assigned remodelling stage labels as inputs. All artery morphology feature values were standardized (mean subtracted and divided by the standard deviation) and all arteries were used as the training data. The LDA output was as follows (Supplementary Table 3): the optimal linear combination of a subset of features that maximized the separation by manual stage between arteries in LDA space; and the coordinates of each artery in LDA space.

To define the SAR trajectory, a fourth-degree polynomial was fitted to the artery coordinates in LDA space. To determine the optimal degree of the polynomial, polynomials with degrees 1–6 were fitted, and the degree that minimized the *P* value for separating *δ* distributions between arteries grouped using the manual remodelling stage (Extended Data Fig. 4c) was selected. The polynomial fit was implemented using the MATLAB function fit and resulted in the following polynomial: *f*(*x*) = 0.0005 × *x*^4^ – 0.01227 × *x*^3^ + 0.1363 × *x*^2 ^– 0.4354 × *x* – 0.7425. The polynomial was then numerically interpolated on a dense 10^4^-point grid, and the distance from each artery point in LDA space to the polynomial was calculated using this grid and the MATLAB exchange function distance2curve^64^. *δ* per artery was then calculated as the line integral from the curve origin to closest point to the artery on the curve (Extended Data Fig. 4a, inset). This integral was numerically calculated using a custom MATLAB script. *δ* values were linearly rescaled to the range 1–5 using the MATLAB function rescale.

### Cell-type frequency as a function of GA and SAR

To examine cell-type frequencies within the decidua as a function of GA and SAR (Figs. 3 and 4), per-image cell frequency tables were constructed in which cell-type frequencies were calculated as the proportion of cells in the decidua feature mask of that image. Cells located in other feature masks (artery, gland, vessel or cell column masks) were not counted, nor were cells of an unassigned type (‘other’). To focus these analyses on cell populations strictly found in the decidua, muscle and glandular cells were also excluded; these cell types occasionally extended outside their artery and gland feature masks, respectively. Cell frequency as a function of GA for a cell type was defined as the per-image proportion values for that cell type, as a function of the GAs associated with the images. Similarly, cell frequency as a function of SAR for a cell type was defined as the per-image proportions of that cell type, as a function of the mean *δ* values per image. For the volcano plot in Fig. 3n, we fitted a linear regression model to the two above-described functions. All linear regression models were implemented using the MATLAB function fitlm and the volcano plot only shows points for which regression *R*^2^ ≥ 0.05. *R*^2^ and *P* values for all *δ*-based and GA-based regressions are provided in Supplementary Table 13. The ratio between *R*^2^ in the two regression models was used to classify trends as GA-driven, SAR-driven or synchronized. For example, the increase in EVTs out of all cells, R_EVT, was classified as GA-driven because *R*^2^ for R_EVT as a function of *δ* was 0.3 but only 0.1 for R_EVT as a function of GA (Extended Data Fig. 4d and Supplementary Table 10). Another example is the increase in macrophages out of immune cells, I_sumMac: it was classified as GA-driven because *R*^2^ for I_sumMac as a function of GA was 0.6 but only 0.1 for I_sumMac as a function of *δ* (Extended Data Fig. 4e and Supplementary Table 10). To determine the trend sizes depicted in Fig. 3n, the following calculation was used: denote the per-image frequencies of a cell type as *V*, and the corresponding per image temporal stamps (either GA or mean image *δ*) as *X*. Trend size is then calculated as the difference between the first and last time point in units of the mean: $\frac{V(\max (X))-V(\min (X))}{{\rm{mean}}(V)}$.

### NanoString GeoMx DSP

The experiment was performed using NanoString Technologies according to company manuals, details are below.

#### Slide preparation

Serial sections of the TMAs were cut into 5 µm FFPE sections and were mounted on SuperFrost Plus slides (Fisher Scientific, 12-550-15), air dried and baked overnight at 60 °C. Slides were then processed as specified by the NanoString GeoMx DSP Slide Preparation User Manual (NanoString Technologies, MAN-100 7). In brief, slides were dewaxed, underwent antigen retrieval and treated with proteinase K (Ambion, 2546) at 1 µg ml^–1^ concentration. Slides were then post-fixed. For RNA probe hybridization, slides were placed in a slide rack with Kimwipes damped with 2× SSC lining the bottom. Each slide was treated with 200 µl of NanoString Technologies whole transcriptome RNA probe mix at a concentration of 4 nM per probe in 1× buffer R (NanoString Technologies). A Hybridslip (Grace Biolabs, 714022) was applied over each slide. Slides were incubated at 37 °C overnight. After hybridization, slides were dipped in a 2× SSC with 0.1% Tween 20 (Teknova, T0710) to remove the coverslips. They were then washed twice in 2× SSC and 50% formamide (ThermoFisher AM9342) at 37 °C for 25 min followed by two washes in 2× SSC for 5 min each at room temperature. Slides were blocked in buffer W (NanoString Technologies) at room temperature for 30 min, followed by the application of 200 µl morphology marker mix for 1 h. Details of the morphology markers are provided in Supplementary Table 7.

#### Sample collection

Sample collection was performed as indicated in the GeoMx DSP instrument user manual (MAN-10088-03). Slides were loaded into the GeoMx DSP instrument and scanned. For each tissue sample, we selected ROIs corresponding to one of the following categories: artery (13), decidua (13), interstitial EVT (5), intravascular EVT (3); in total, 34 ROIs were selected (Supplementary Table 11). Morphology markers for SMA and VIM were used in conjunction with a serial H&E section to provide tissue context and to locate arteries and decidua on the platform. Artery, decidua and intravascular EVT ROIs were selected using the geometric selection tool, and interstitial EVTs were selected using a HLA-G^+^ mask. Intravascular EVTs were identified as HLA-G^+^ cells located within arteries. Each ROI was collected into a single well in a 96-well plate.

#### GeoMx DSP NGS library preparation and sequencing

Each GeoMx sample or well was uniquely indexed using an i5 × i7 dual-indexing system from Illumina. In total, 4 µl of a GeoMx DSP sample was used in a PCR reaction with 1 µM of i5 primer, 1 µM i7 primer and 1× NSTG PCR master mix. For the PCR amplification reaction, each 96-well plate was placed in a thermocycler programmed with the following protocol: 37 °C for 30 min, 50 °C for 10 min, 95 °C for 3 min, 18 cycles of 95 °C for 15 s, 65 °C for 60 s, 68 °C for 30 s, and final extension of 68 °C for 5 min. PCR assays were purified with two rounds of AMPure XP beads (Beckman Coulter) at 1.2× bead-to-sample ratio. Libraries were paired-end sequenced (2 × 75) on a NextSeq550 with up to 400 million total aligned reads.

#### Normalization and scaling of GeoMx counts data

Raw counts from each gene in each sample were extracted from the NanoString GeoMx NGS processing pipeline (Supplementary Table 11). Quality control was done according to the NanoString data analysis manual (MAN-10154-01) with default parameters as indicated in the manual. For each EVT sample, the counts were normalized using one of the manufacturer’s recommended approaches for normalizing GeoMx data: dividing all genes in each sample by the 75th percentile of expression in that sample, followed by multiplication by an identical scaling factor for all samples: the geometric mean of all 75th percentiles. This approach eliminates differences in counts between samples due to ROI-specific properties such as size and RNA-binding efficiency. The background due to nonspecific binding per sample was approximated with the geometric mean of the 100 negative control probes included in the probe mix, as recommended by NanoString Technologies. The above-described normalization step eliminated the correlation between background and ROI size for EVT samples. For artery and decidua samples, normalization was complicated by the fact that the ROI size was tightly correlated with SAR stage and therefore biologically meaningful trends in the data. This led to the correlation between ROI size and background not being entirely eliminated by normalization. We therefore used a background subtraction correction technique before normalization as recommended in the NanoString Technologies manual for such cases. The correction was performed by subtracting the geometric mean of negative probes from gene counts on a per-sample basis and proceeding with normalization as previously described.

### Gene expression in artery as a function of GA and SAR

In brief, for each gene, we performed polynomial regressions of gene expression with *δ* and GA as the independent variables and used regression *P* values to determine which genes were trending and the ratio of regression *R*^2^ values to classify the trends as detailed below.

The NanoString Technologies RNA probes panel contains probes for 18,696 transcripts. For this analysis on artery samples, only genes with background-subtracted, normalized counts ≥10 in at least two arteries were considered. This resulted in 14,471 expressed genes. Each artery sample was assigned a remodelling score *δ* based on the *δ* of the sampled artery in the MIBI data. If several arteries were sampled, the assigned *δ* was the average *δ* values of the sampled arteries. Endothelial loss and SMA loss per sample were calculated similarly based on the corresponding MIBI values (Supplementary Table 11). The following steps were then performed on artery samples.

For all expressed genes, gene expression as a function of GA was defined as the background-subtracted and normalized counts for that gene, as a function of the GAs associated with the samples. Similarly, expression as a function of SAR for a gene was defined as the per-sample background-subtracted and normalized counts of that gene, as a function of the *δ* values per sample. A second-degree polynomial regression model was then fitted to the two above-described functions. The reason for using a second-degree polynomial instead of linear regression was to allow the regression models to capture non-monotonic trends in gene expression. All regression models were implemented using the MATLAB function fitnlm. Expression fold change was defined as the ratio between the maximum and the minimum of expression values. The centre of mass (COM) of the expression trajectory of a gene as a function of *t* (*t* being either GA or *δ*) was defined as the weighted mean of *t* values, where the weights are the expression values at the respective *t*.

Genes with a *P* value ≤ 0.05 and fold change ≥2 for either GA or *δ* regression were classified as trending genes. The ratio between *R*^2^ in the two regression models was used to classify trending genes as GA-driven, SAR-driven or synchronized. Trending genes with ${\log }_{2}({R}_{\delta }^{2}/{R}_{{\rm{GA}}}^{2})\ge 1$ and ${R}_{\delta }^{2}\ge 0.05$ were classified as SAR-driven, whereas genes with ${\log }_{2}({R}_{\delta }^{2}/{R}_{{\rm{GA}}}^{2})\le 1$ and ${R}_{{\rm{GA}}}^{2}\ge 0.05$ were classified as GA-driven. Other trending genes were classified as synchronized (Supplementary Table 4).

For visualization only, two fitted expression trajectories (one as a function of GA and another as a function of *δ*) were calculated per gene. These fitted expression trajectories were calculated as the values of the fitted second-degree polynomial model at five evenly spaced values of GA and *δ*, respectively. To compare fitted expression trajectories between genes, they were normalized by *Z*-scoring their value per gene (Fig. 3o).

See Supplementary Information for further details about analysis of NanoString data in decidua ROIs.

### Coordinated gene expression by pathways in the artery

We set out to find gene pathways with coordinated expression trends among our genes of interest: genes trending with *δ* in arteries. To find these coordinated pathways, we first defined the pathways and then defined temporal coordination.

To define pathways, we used the R package msigdbr to obtain the lists of genes per pathway for the Gene Ontology by Biological Process database (7,481 pathways). We then cross-referenced the list of genes for each pathway with the genes of interest and discarded pathways with an intersection of fewer than ten genes. For the remaining pathways, we examined whether the pathway genes that appeared in the gene set of interest exhibited coordination in their expression as function of *δ*.

A group of coordinated genes was defined as a group of genes for which the COMs were significantly closer to each other than one would expect at random (see previous section for the definition of COM). Using the spread of COMs as a measure for coordination allowed us to leverage the raw data rather than fitted gene expression trajectories while still maintaining robustness against noise.

To calculate the extent of coordination between a group of *N* genes, we first calculated their median COM, denoted COM_med_. Then, their COM dispersal was defined as the median of the absolute deviations from COM_med_ for the *N* genes, denoted CD. To determine whether the CD for the gene group, CD_group_, is significantly smaller than expected at random, we calculated the randomly expected CD, denoted CD_rand_. This was done by selecting *N* random genes without replacement and calculating their CD, 10^5^ times to estimate the null distribution. The random CD_rand_ was then calculated as the median over the CDs for randomized gene sets. The coordination score for our *N* genes group was then defined as log_2_(CD_rand_/CD_group_). The *P* value for the coordination score was defined as the number of times a randomized CD was smaller than CD_group_, divided by the number of randomizations (10^5^). (1/number of randomizations) was then added to all *P* values to account for the finite number of randomizations. *q* values were calculated using the Benjamini and Hochberg method on *P* values, implemented using the MATLAB function mafdr.

The CD, coordination scores, *P* values and *q* values were calculated as described above for all 7,481 pathways. Pathways with coordination score ≥ 1.5 and *P* value ≤ 0.05 were considered to be coordinated (Supplementary Table 4).

### Ridge regression for predicting GA from immune composition

Ridge regression was implemented using the sklearn Python package (sklearn.linear_model.Ridge, RidgeCV). Per-image immune frequencies were rescaled to the range 0–1 before model fitting using the sklearn scaling function. Images with fewer than ten immune cells were excluded (*n* = 8). A randomly derived test–train split of 30/70 was used, and GA distribution was verified to be equally represented in the test and train sets (Extended Data Fig. 6a). Ridge regression adds a regularization penalty to the loss function to prevent over or under representation of correlated variables, such as immune cell populations. The penalty used for the test set (0.81) was selected using leave-one-out cross-validation on the training set.

### Cell–cell and cell–artery spatial enrichment analysis

To identify preferential colocalization of maternal immune cells in decidua, we measured the spatial proximity enrichment for all cell-type pairs, which evaluates the spatial organization of cell types relative to each other, as previously described^22–24^. Cells located in non-decidual feature masks (artery, gland, vessel or cell column masks) were not included in this analysis. The distances in pixels between all pairs of cells were calculated in each image. The resulting per-image distance matrices were binarized with a distance threshold (100 pixels or 39 μm in our case), and pairs of cells closer than 100 pixels from each other were considered a close interaction. To evaluate the number of close interactions between two cell types, this proximity matrix was subset column-wise by cell type A and subset row-wise by cell type B. The sum of the resulting submatrix quantified the number of close interactions between the cells of types A and B. To evaluate the significance of the number of close interactions, given the total number of cells in the image, tissue architecture and composition across the cohort, and total number of cells of types A and B in the image, a bootstrapping approach was used. For each of 100 bootstrapping iterations, the location of cells of type A was randomized across all cell locations (of any type) in the image while their total number was preserved. The number of close interactions with cells of type B was calculated for each randomized iteration. Repetitions of this process approached a null distribution for the number of close interactions between cells A and B. The enrichment score for cells A around cells B in the image was then calculated as the *Z*-score of the measured number of close interactions between A and B when *Z*-scored together with the random bootstraps. This analysis was extended to incorporate enrichment of cell types around spiral arteries. For each cell, the distance to the nearest spiral artery was considered. An additional column was added to the proximity matrix described above, which thresholded distances between cells and arteries with the same 100 pixel threshold. The above-described bootstrapping approach also provided a null distribution for artery proximity. Tools for this analysis were written in Python, with the bootstrapping accelerated using Cython. An intuitive, easy-to-use Jupyter Notebook interface was created to allow for easy implementation of this algorithm. For per-image spatial enrichment scores, see the Data availability statement. The code for this analysis is available at GitHub (https://github.com/angelolab/ark-analysis).

### Cell–cell and cell–artery enrichment temporal trends and trending with GA or SAR or constant

For examining cell–cell and cell–artery enrichment within the decidua as a function of GA and SAR (Extended Data Figs. 6c and 2c), per-image enrichment score matrices *E* were calculated as described in the previous section, in which *E*_*i,j*_ is the enrichment score of cell type *i* around cell type *j* in the image. Enrichment as a function of GA was defined as the per-image enrichment, as a function of the GAs associated with the images. Similarly, enrichment as a function of SAR was defined as the per-image enrichment, as a function of the mean *δ* values per image. We fitted a linear regression model to the two above-described functions. All linear regression models were implemented using the MATLAB function fitlm. *R*^2^ and *P* values for all *δ*-based and GA-based regressions are provided in Supplementary Table 3. The ratio between *R*^2^ in the two regression models was used to classify trends as GA-driven, SAR-driven or synchronized like in Fig. 3n. Extended Data Fig. 6c only shows points for which regression *R*^2^ ≥ 0.05, *P* value ≤ 0.05, maximal absolute value of linear fit ≥ 2. Trends including muscle, fibroblast, myofibroblast, glandular, other and endothelial cells were not considered in this analysis. For determining trend sizes, the following calculation was used: denote the linear fit to per-image enrichment scores as *V*, and the corresponding per-image temporal stamps (either GA or mean image *δ*) as *X*. Trend size is then calculated as (*V*(max(*X*)) – *V*(min(*X*))).

To determine whether two cell types were significantly enriched around each other throughout the cohort, we averaged their pairwise enrichment over all images. The pair was considered enriched if the absolute value of mean enrichment was ≥2 (Supplementary Table 3).

In Extended Data Fig. 6c, the following cell–cell enrichments were not plotted for clarity: enrichment of a cell type around itself (for example, T_reg_ cells around T_reg_ cells); enrichments including muscle, fibroblast, myofibroblast, glandular, other and endothelial cell types; enrichment trends that are SAR-driven.

### Functional marker positivity rate per cell type as a function of GA and SAR

For examining cell-type-specific temporal trends in the expression of functional markers (Fig. 5a), 48 combinations of cell-type functional marker were selected. The selected combinations were those for which the positivity frequency *Z*-score exceeded 0.5 (Fig. 2a, right). For each of these combinations, the frequency of cells positive for the functional marker was calculated as the number of cells positive for the marker (see the methods section ‘Definition of thresholds for functional marker positivity’) out of the total number of cells of the same cell type in the image. All cells except those located within the cell column mask were included to focus the analysis on functional marker trends of maternal cells and EVTs that had infiltrated the decidua. For glandular cells, the location was further restricted to the glands mask. The frequency of cells positive for a functional marker as a function of GA, for a cell type, was defined as the per-image positivity proportion values as a function of the GAs associated with the images. Similarly, marker positivity frequency as a function of SAR for a cell type was defined as the per-image proportions of that cell type positive for the marker, as a function of the mean *δ* values per image. For the volcano plot in Fig. 5a, we fitted a linear regression model to the two above-described functions. All linear regression models were implemented using the MATLAB function fitlm, and the volcano plot only shows points for which regression *R*^2^ > 0.05. *R*^2^ and *P* values for all *δ*-based and GA-based regressions are provided in Supplementary Table 10. For determining trend sizes depicted in Fig. 5a, the following calculation was used: denote the linear fit to the per-image marker positivity proportion of a cell type as *V*, and the corresponding per-image temporal stamps (either GA or mean image δ) as *X*. Trend size is then calculated as the difference between the first and last time point in units of the mean: $\frac{V({\rm{\max }}({\rm{X}}))-V({\rm{\min }}(X))}{{\rm{mean}}(V)}$.

### Cellular microenvironments

For each cell in the dataset, we defined a ‘neighbourhood’ consisting of its 25 closest neighbouring cells, as measured by Euclidean distance between *x*/*y* centroids, excluding cells that were not in the decidua (that is, cells that overlapped with any artery, gland, anchoring villous or vessel feature masks). We clustered these cellular neighbourhoods on the basis of their composition of the 26 cell populations as identified previously using FlowSOM. For clustering, we used the scikit-learn implementation of *k*-means algorithm with *k* = 20 to identify neighbourhoods characterized by the presence of rare cell populations. Selected clusters were merged on the basis of similarity when hierarchically clustered, a threshold of 0.5 when comparing Euclidean distances between *k*-means cluster centroids and manual inspection of the cluster assignment when overlaid on the images. Based on these approaches, we defined 12 distinct decidual cellular microenvironments, 10 of which are shown in Fig. 5f (microenvironments characterized by predominantly stromal cell populations, fibroblasts (3 in Supplementary Table 12) and myofibroblasts (6 in Supplementary Table 12) are not shown in the heatmap).

### Definition of anatomical EVT location and associated arteries

Cell column EVTs were defined as EVTs located within cell column masks, intravascular EVTs were located within artery masks, and interstitial EVTs were located in the decidua. Perivascular EVTs were defined as interstitial EVTs located within 50 pixels of the edge of an artery, as defined by the radial expansion of the artery masks (Extended Data Fig. 7b). Arteries were said to have perivascular or intravascular EVTs (Fig. 6d and Extended Data Fig. 7e,f) if the number of EVTs in the appropriate artery compartment was >5.

### SMA and endothelium-loss scores

The loss scores presented in Extended Data Fig. 7e,f were based on digitized morphological features. For SMA, the average feature was used, whereas for endothelium, the radial coverage of CD31 was used (see methods section ‘Automated digitization of artery morphological features’). The values for each of the two features were then divided by their maximum across arteries and subtracted from 1 to obtain a loss score. The resulting values were then linearly rescaled to the range 0–1 using the MATLAB function rescale.

### Characterization of EVTs by compartment

To further characterize EVT composition per spatial compartment (cell column, interstitial, perivascular or intravascular), we first quantified EVT-subtype frequency per compartment. One image was excluded (16_31762_20_8) owing to abnormal tissue morphology (Extended Data Fig. 8a). We then compared the distance from the nearest artery between EVT subtypes (Extended Data Fig. 8b). For this analysis, only images that contained all four EVT types were considered, and the cell-to-artery distance was measured from the cell centroid as detected by segmentation to the closest point on the border of the artery mask.

We then set out to assess the extent of similarity between EVTs by compartment in terms of expression of functional markers. For this analysis we used a LDA based method, similarly to our calculation of the continuous SAR remodelling score *δ* for compartment-wise analysis of EVT types. The input table for LDA consisted of MIBI-measured marker expression values per EVT. The following lineage and functional markers expressed by EVTs were included: CD56, CD57, HLA-G, CK7, PD-L1 and Ki67. EVTs were labelled by spatial compartment as cell column, interstitial, perivascular or intravascular. Marker expression values were standardized (mean subtracted and divided by the standard deviation), and cell column, interstitial and intravascular location labels per EVT were used for training the LDA model. Perivascular EVTs were withheld as a test set. Owing to the small number or features (markers), a one-dimensional LDA was calculated to produce a single coordinate, LD1. LD1 was the optimal linear combination of a subset of markers to maximize the separation by compartment between EVTs (Supplementary Table 14). LD1 values were subsequently calculated for the withheld test set of perivascular EVTs (Supplementary Table 14). The distributions of LD1 values per compartment indicated that perivascular EVTs are similar to interstitial and intravascular EVTs, with a median value between the two (Extended Data Fig. 8c). This result implies that perivascular EVTs could be an intermediate state between interstitial and intravascular, in line with the intravasation model whereby interstitial EVTs invade the artery lumen.

### Origin of CD56^+^ EVTs in the intravascular compartment

The frequency of CD56^+^ EVTs was highest in the intravascular compartment (Extended Data Fig. 8a). Furthermore, the frequency of CD56^+^ EVTs increased with SAR both in the perivascular and intravascular compartment (Extended Data Fig. 8d,e). However, the increase in the intravascular compartment was steeper (Extended Data Fig. 8f). We set out to test whether this was compatible with intravasation whereby the source for intravascular EVTs is perivascular EVTs or whether additional sources of intravascular CD56^+^ EVTs were needed to account for their increase. These alternative sources could be cell proliferation or extravasation, whereby EVTs migrate in a retrograde manner after entering spiral arteries directly at the basal plate. Under the intravasation model, the steep increase in CD56^+^ EVTs between the perivascular and intravascular compartments would be explained by CD56 upregulation upon arterial invasion. We proposed that this should involve an intermediate state of perivascular EVTs defined by moderate levels of CD56. To test this hypothesis, we compared the average CD56 intensity of perivascular and intravascular EVT1a and EVT1b cells for each artery (for arteries that initiated remodelling: *δ* ≥ 2). This analysis detected a significant increase in CD56 expression between the perivascular and intravascular compartment by EVT1a and EVT1b cells (sided Wilcoxon signed rank test *P* value = 5 × 10^−3^; Extended Data Fig. 8g). An alternative explanation for the increasing frequency of CD56^+^ EVT1c cells within arteries could be proliferation. However, only 0.5% of intravascular EVT1c cells were Ki67^+^, a lower frequency than 9.6% and 1.8% for intravascular EVT1a and EVT1b cells, respectively, which suggested that proliferation is not a primary contributor (Extended Data Fig. 8h).

### DEGs in EVTs

DEGs between intravascular and interstitial EVTs were identified using the Bioconductor package limma^65^ (linear models for microarray data) after consulting with the NanoString statistics team. Using the default parameters in limma on 75th percentile normalized counts, 131 upregulated genes and 143 downregulated genes were found (false-discovery rate cutoff = 0.1, log fold change cutoff = 2). Genes with log fold change ≥ 2.3 or ≤ 2.3 and adjusted *P* value ≤ 0.05 are shown in the heatmap in Fig. 6f. A complete list of DEGs is shown in Extended Data Fig. 9 and Supplementary Table 11. For IHC validation of differential expression for selected genes, see Extended Data Fig. 10a.

### NicheNet analysis

We used the NicheNet R package to predict ligand–receptor interactions between intravascular EVTs and arteries. The analysis was performed by following the vignette available at GitHub (https://github.com/saeyslab/nichenetr/blob/master/vignettes/ligand_activity_geneset.md).

NicheNet requires three input gene lists to predict ligands in sender cells that are likely to interact with receptors in receiver cells and by doing so affect the expression of a set of genes of interest. These three gene lists are: genes of interest, genes expressed in sender cells and genes expressed in receiver cells.

For our analysis, we wanted to check which ligands expressed in intravascular EVTs are likely to be causing temporal gene expression trends with remodelling in arteries. To do so, we defined the genes of interest as all genes trending with remodelling in arteries (Fig. 3o). The genes expressed in receiver cells were defined as all genes expressed in arteries (see previous sections for the definition of ‘expressed’), and genes expressed in sender cells were defined as genes differentially expressed between interstitial and intravascular EVTs and higher in intravascular EVTs (Supplementary Table 11). NicheNet analysis was performed as described in the vignette to prioritize ligands and to infer corresponding receptors and downstream targets (Extended Data Fig. 10b). The inferred targets were manually classified according to their known function using the Gene Cards database (https://www.genecards.org) and survey of the literature. A list of references for all classifications are provided in Supplementary Table 5.

### Statistical analyses

Throughout the paper, unless indicated otherwise, the Kruskal–Wallis test was used. It was implemented using the MATLAB function KruskalWallis. All linear regression models were implemented using the MATLAB function fitlm unless stated otherwise. The sided Wilcoxon signed-rank test for paired analysis was implemented using the MATLAB function signrank. MATLAB v.2020b was used throughout the article for statistical analysis.

### Ethics statement

All human samples were acquired and all experiments were approved by Institutional Review Board protocol number 46646 “Assessing normal expression patterns of immune and non-immune markers across tissue types with multiplexed ion beam imaging” at Stanford University. Per this protocol from the Institutional Review Board at Stanford University, the consent to use archival deidentified tissue was not required. All experiments followed all relevant guidelines and regulations.

### Reporting summary

Further information on research design is available in the Nature Portfolio Reporting Summary linked to this article.

## Online content

Any methods, additional references, Nature Portfolio reporting summaries, source data, extended data, supplementary information, acknowledgements, peer review information; details of author contributions and competing interests; and statements of data and code availability are available at 10.1038/s41586-023-06298-9.

## Supplementary information

Supplementary Information Full legends for Supplementary Tables 1–14, and Supplementary Methods.

Reporting Summary

Supplementary Table 1 Patients and block histology. See Supplementary Information PDF for full legend.

Supplementary Table 2 Artery properties and staging.

Supplementary Table 3 Cell–artery and cell–cell spatial enrichment.

Supplementary Table 4 NanoString DSP analysis of artery ROIs.

Supplementary Table 5 NicheNet analysis.

Supplementary Table 6 Preeclampsia indicated genes.

Supplementary Table 7 Antibodies.

Supplementary Table 8 Number of segmented events per image.

Supplementary Table 9 Positivity binary threshold for functional markers.

Supplementary Table 10 Volcano plots for Figs. 3 and 5.

Supplementary Table 11 NanoString DSP counts and meta-data.

Supplementary Table 12 Cell microenvironment lineage composition.

Supplementary Table 13 NanoString DSP analysis of decidua ROIs.

Supplementary Table 14 EVT by compartment LDA analysis.

## Data Availability

MIBI data are available at the Human BioMolecular Atlas Program (10.35079/hbm585.qpdv.454). The same MIBI data in a browsable format, along with segmentation masks, extracted features, cell phenotype masks (CPMs), cell–cell and cell–artery spatial-enrichment scores per image, a table enumerating all single cells in this study and provides their location, morphological characteristics (such as size and shape), marker expression, FlowSOM cluster assignment and cell-type assignment, are available at FigShare (10.6084/m9.figshare.16663465). H&E images of tissue blocks from the cohort with annotations are available at Dryad (10.5061/dryad.v15dv41zp). The following public databases were used in this study (see Methods for details): the Gene Ontology Biological Process database (http://geneontology.org/) and the Gene Cards database (https://www.genecards.org/). Source data are provided with this paper.

## Source data

Source Data Fig. 1
Source Data Fig. 2
Source Data Fig. 3
Source Data Fig. 4
Source Data Fig. 5
Source Data Fig. 6
Source Data Extended Data Fig. 2
Source Data Extended Data Fig. 3
Source Data Extended Data Fig. 4
Source Data Extended Data Fig. 5
Source Data Extended Data Fig. 6
Source Data Extended Data Fig. 7
Source Data Extended Data Fig. 8
Source Data Extended Data Fig. 9
Source Data Extended Data Fig. 10

## Code availability

The following software were used for analysis in the article: ImageJ, MAUI (https://github.com/angelolab/MAUI) for low-level image processing; DeepCell v.0.5.0 (https://deepcell.readthedocs.io/en/master/index.html) for cell segmentation; Ark Analysis for cell–cell spatial enrichment (https://github.com/angelolab/ark-analysis); and QuPath (v.0.4.0) for two-colour IHC analysis. Custom code for this study is available at FigShare (10.6084/m9.figshare.16663465).
