# Supplementary material for: A spatially resolved timeline of the human maternal–fetal interface
Source: Nature. 2023 Jul 19;619(7970):595–605. doi: 10.1038/s41586-023-06298-9 (PMC10356615; doi:10.1038/s41586-023-06298-9)
Supplement: Supplementary file 1 — Full legends for Supplementary Tables 1–14, and Supplementary Methods. [file 41586_2023_6298_MOESM1_ESM.pdf]

nature portfolio

https://doi.org/10.1038/s41586-023-06298-9

Supplementary information

A spatially resolved timeline of the human maternal–fetal interface

In the format provided by the authors and unedited

Supplementary Information

Contains Supplemental Tables Guide and Supplemental Methods regarding antibody validation, cell segmentation and phenotyping, decidual ROIs Nanostring data analysis and immunoregulatory marker expression. Including 4 Supplementary Information Figures titled:

1. MIBI-TOF antibody panel
2. Workflow for Mesmer whole-cell segmentation of single cells from MIBI images and cell phenotyping
3. Decidual ROIs Nanostring data analysis
4. Single-cell expression of immunoregulatory markers

Table of Contents

***Supplementary Tables Guide* 2**

***Supplementary Methods* 5**

Antibody Validation 5

Cell Segmentation and Clustering 6

Analysis of NanoString Whole Transcriptome Data for Decidua ROIs 15

Tim-3 and Galectin-9 Co-expression in single cells 17

Supplementary Tables Guide

Supplementary Table 1 - Patients and block histology. First tab- Patient meta-data such as age, ethnicity, body mass index, parity and relevant medical conditions such as HIV. Second tab- Information on the histological characteristics of the blocks retrieved, including the presence of cell column anchoring villi. Per patient's block, indication whether cell column anchoring villi were present (1) or absent (0), and the number of regions containing spiral arteries annotated as appropriate for TMA construction by the pathologist (Methods). In blocks containing ≥ 2 distinct, separate pieces of tissue, cell column villi were considered present if they were present on any piece containing pathologist annotations.

Supplementary Table 2 - Artery properties and staging. First tab- Arteries meta-data, including their measured digitized morphological features (see Methods), manual stage and remodeling score δ. Second tab- LDA coefficients for artery morphological features. Per feature LDA coefficients (ld1,ld2) and their Z scored absolute values.

Supplementary Table 3 – Cell-artery and cell-cell spatial enrichment. First tab- Linear regression results of cell types enrichment around arteries as function of remodeling score δ. Rows: cell types. Columns: regression R^2, p-value, maximal absolute value of enrichment and trend size (see Methods). Second tab - temporal trends in cell-cell enrichment. Rows: combinations of 2 cell types in the format: cell type a- cell type b. The information in the row is for the enrichment of cell type a around cell type b. The columns show values for the linear regression on per image basis of enrichment scores as function of GA and remodeling score δ: R^2, the maximal obtained regression R^2, p-value, maximal enrichment (absolute value) and trend size (see Methods). Third tab- constant in time cell-cell enrichment.

Supplementary Table 4 – NanoString DSP analysis of artery ROIs. First tab- Synchronized expression of GO-BP pathways. See Methods for details about p-value calculation. Second tab- Per gene second degree polynomial regression results: R^2, p-values and fold change of gene expression as function of GA and δ.

Supplementary Table 5 – NicheNet analysis. First tab- Comprehensive list of all NicheNet predicted target genes in artery (see Methods) with function classification and references for classification. Second tab- Genes Of Interest (GOI) list : SAR trending genes in artery. Third tab: genes expressed in receiver cells – all genes expressed in artery. Fourth tab- genes in sender cells: DEG between intravascular and interstitial EVTs, higher in intravascular. See methods.

Supplementary Table 6 – PE indicated genes. Genes differentially expressed in decidua samples from women diagnosed with preeclampsia. List taken from PMID: 28618048.

Supplementary Table 7 - Antibodies. First tab- MIBI antibodies: metal reporters, staining concentrations, and parameters used for low-level processing of MIBI images.

Per marker antibody information, including: clone, vendor, vendor ID, channel and elemental reporter, and final staining titers used. The parameters used for marker-specific low-level processing of MIBI data (background removal, denoising, and aggregate removal steps as previously described) are also shown. Second tab- Nanostring morphology markers information**.** Per marker antibody information including clone, channel and final staining titers used.

Supplementary Table 8 – Number of segmented events per image. Number of single cells per image, after segmentation, post processing and exclusion of small non-cellular objects. See Methods.

Supplementary Table 9 - Positivity binary threshold for functional markers. Binary expression thresholds per functional marker- used to determine whether cells are positive for that marker (See methods).

Supplementary Table 10 – Figure 3 and Figure 5 volcano plots. First tab- Values plotted in Fig. 3n. Each row represents a cell proportion with those starting with R_ indication proportion out of all cells in the image, I_ - proportion out of immune cells in image, N_- proportion out of NK cells in image, M_ proportion out of macrophages in image, T_- proportion out of T cells in image, F_- proportion out of EVT in image, S_ proportion out of structural cells in image (Fig. 2b). The columns show values for the linear regression on per image proportions as function of GA and remodeling score δ: the log transformed ratio of R^2, the maximal obtained regression R^2 (maximal between GA and δ), the minimal obtained regression p-value and trend size (see Methods). Second tab- Values plotted in Fig. 5a. Each row represents a combination of a cell type and a functional marker. The columns show values for the linear regression on per image marker positivity rates for the marker-cell type as function of GA and remodeling score δ: the log transformed ratio of R2, the maximal obtained regression R2 (maximal between GA and δ), the minimal obtained regression p-value and trend size (see Methods).

Supplementary Table 11 – NanoString DSP counts and meta-data. First tab- ROI meta-data and raw counts. Meta-data for ROIs. Second tab- mapping Nanostring ROIs to their matching MIBI images. Third tab- raw counts data per ROI. Fourth tab- Differentially expressed genes between interstitial and intravascular EVTs. p-values calculation and multiple hypothesis correction performed by the R Limma package, See methods.

Supplementary Table 12 – Cell microenvironment lineage composition. Fraction of cells from each lineage, for each of the 12 cell microenvironments. See Methods.

Supplementary Table 13 – NanoString DSP analysis of decidua ROIs. First tab- Synchronized expression of GO-BP pathways. See Methods for details about p-value calculation. Second tab- Decidua ROIs deconvolution results using CIBERSORTx. Third tab- match between deconvolved cell types and MIBI cell types. Fourth tab- Per gene second degree polynomial regression results: R^2, p-values and fold change of gene expression as function of GA ,δ and tissue composition.

Supplementary Table 14 – EVT by compartment LDA analysis. First tab- ld1 values per single EVT, LDA input- standardized marker expression values, additional metadata such as EVT type, anatomical location, the image the cell was taken from. Second tab- LDA Coefficients per EVT expressed marker.

Supplementary Methods
Antibody Validation

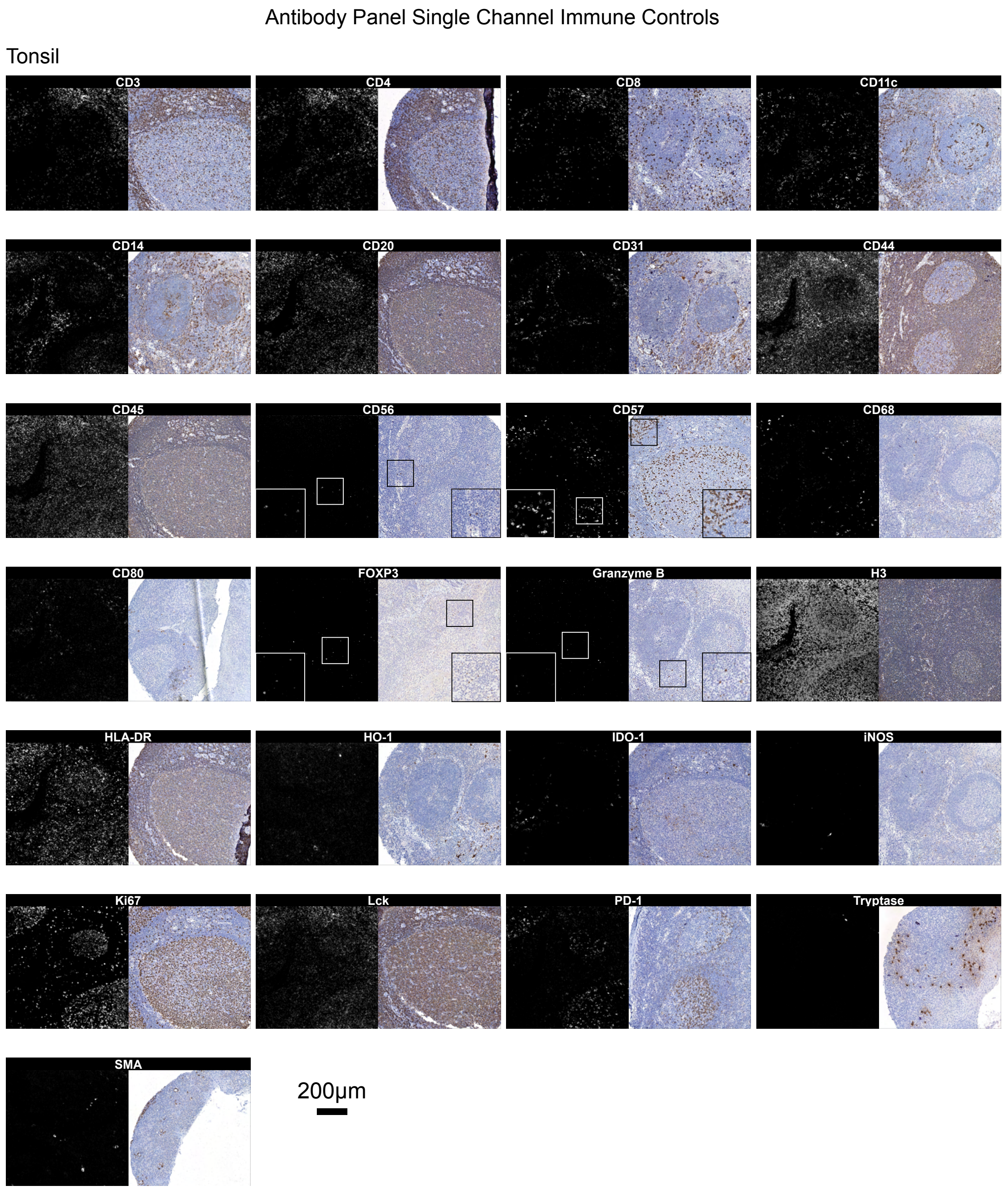

Placenta/decidua

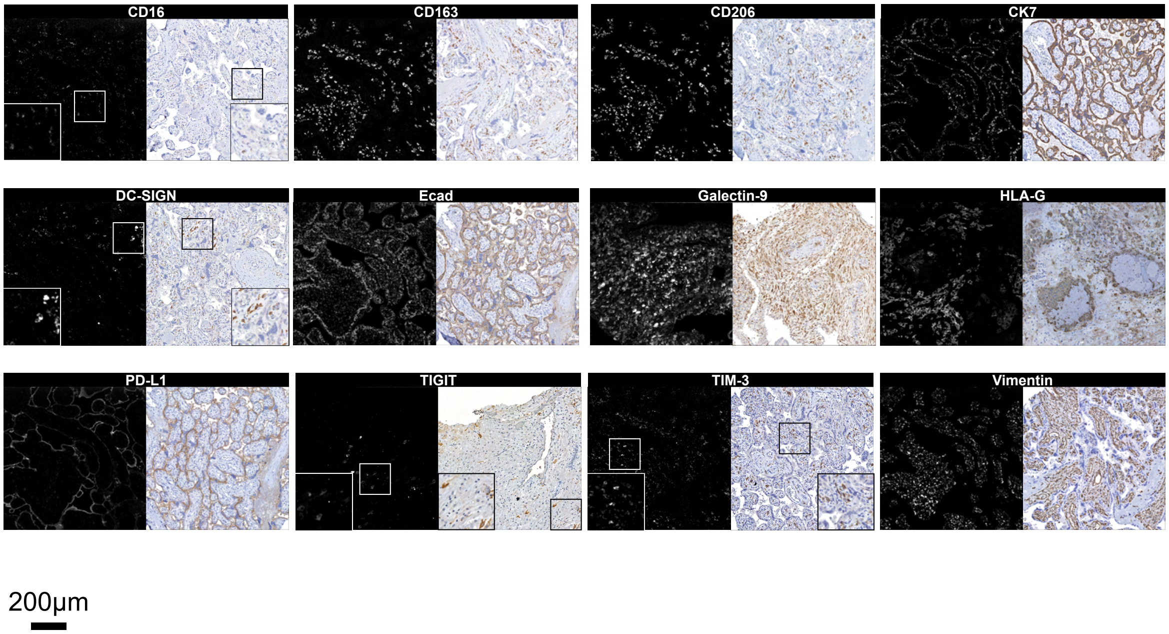

Supplementary Information Figure 1 | MIBI-TOF antibody panel. Representative images of MIBI conjugate staining for all markers along with serial single channel immunohistochemistry images, with immune control tissues (tonsil and placenta). Representative images of n=3. Scale bar, 200 μm.

Cell Segmentation and Clustering

This section contains a figure panel illustrating the single cell segmentation process in greater detail (Supplementary Information Figure 2a), several figure panels illustrating cell clustering was not biased by sample (Supplementary Information Figure 2b-g) and example MIBI images showcasing cells positive for lineage markers (Supplementary Information Figure 2h).

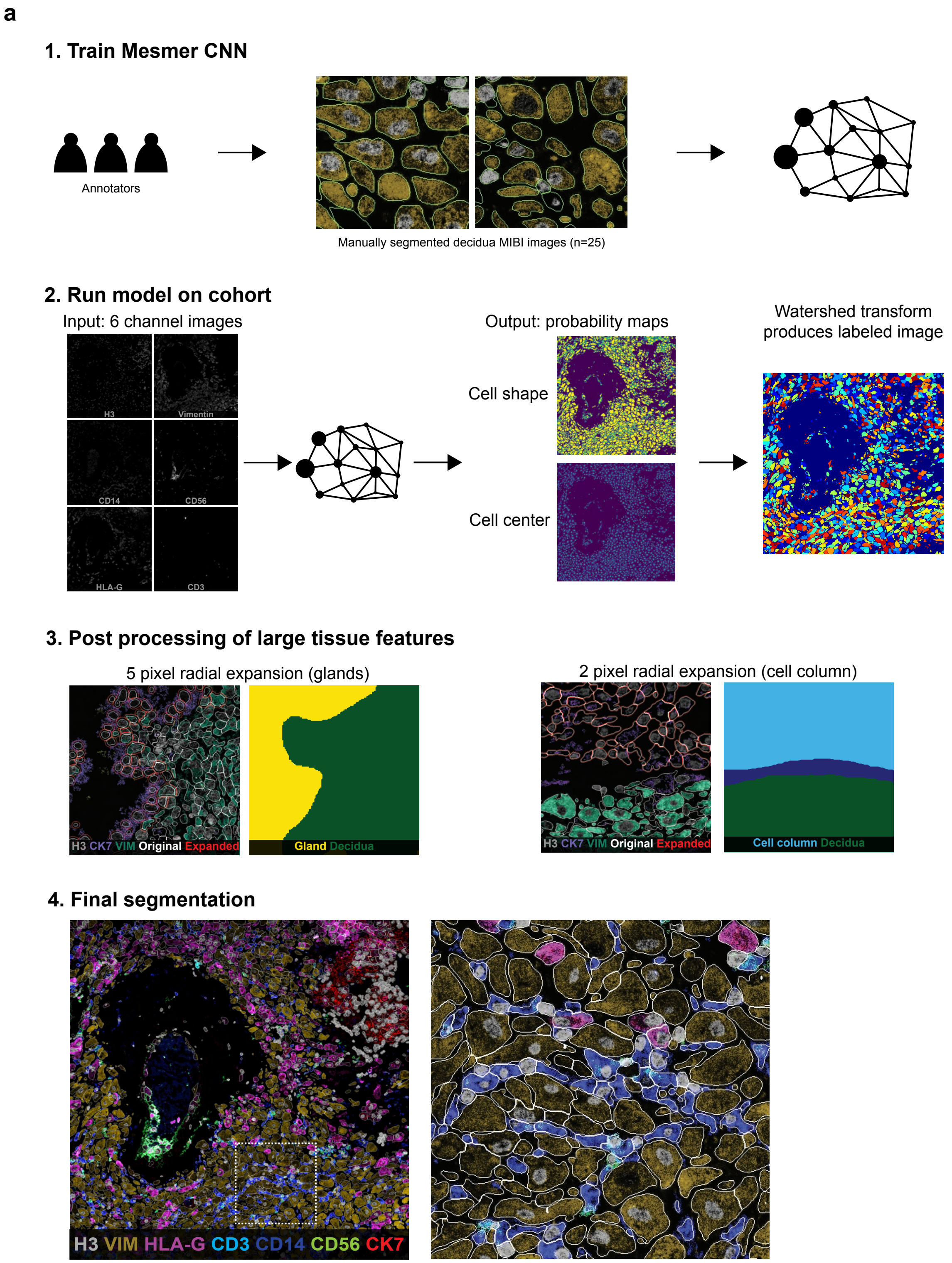
a
1. Train Mesmer CNN
Annotators
Manually segmented decidua MIBI images (n=25)
2. Run model on cohort
Input: 6 channel images
H3
Vimentin
CD14
CD56
HLA-G
CD3
Output: probability maps
Cell shape
Cell center
Watershed transform produces labeled image
3. Post processing of large tissue features
5 pixel radial expansion (glands)
H3 CK7 VIM Original Expanded
Gland Decidua
2 pixel radial expansion (cell column)
H3 CK7 VIM Original Expanded
Cell column Decidua
4. Final segmentation
H3 VIM HLA-G CD3 CD14 CD56 CK7

b

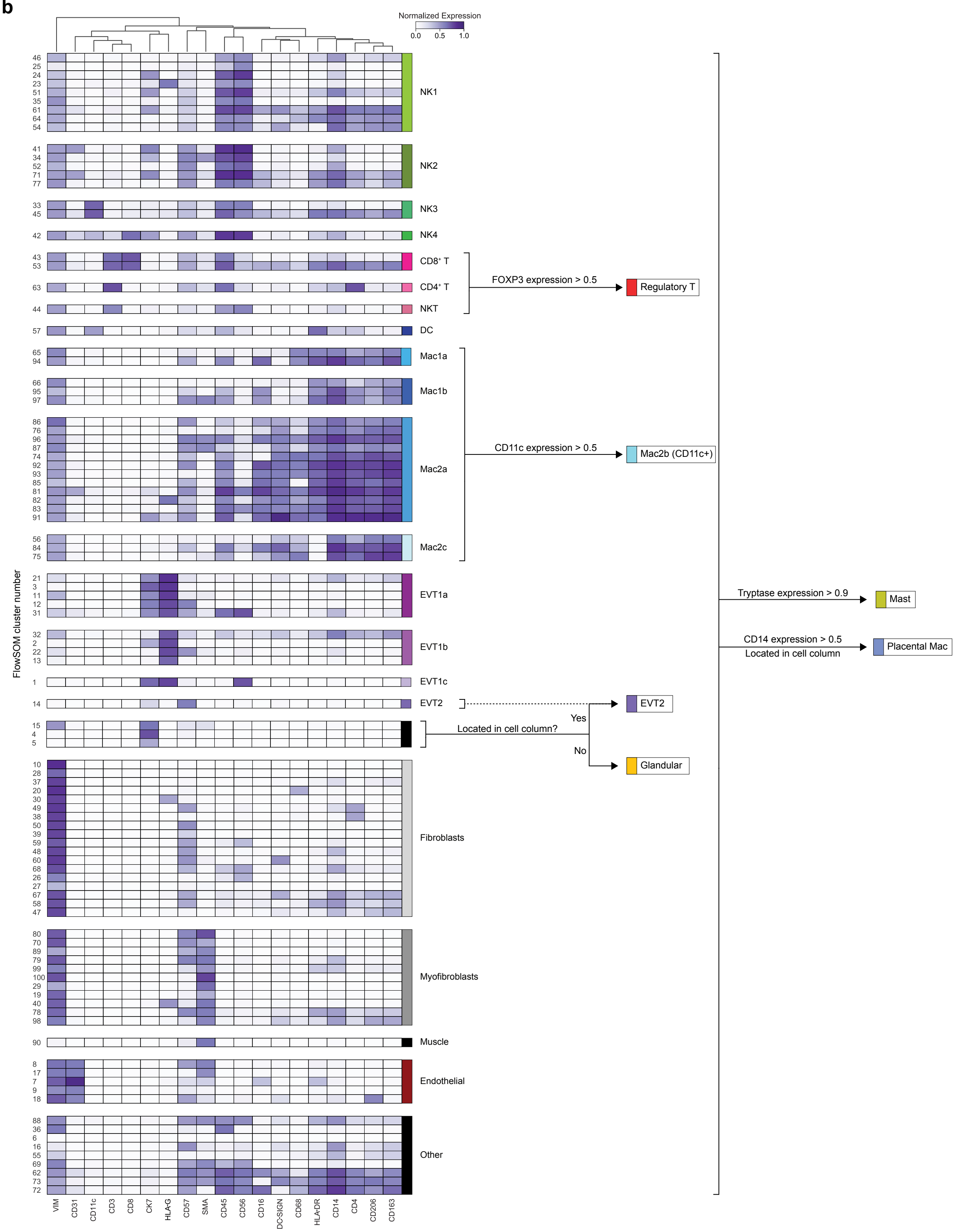
Normalized Expression
0.0
0.5
1.0
FlowSOM cluster number
NK1
NK2
NK3
NK4
CD8⁺ T
CD4⁺ T
NKT
DC
Mac1a
Mac1b
Mac2a
Mac2c
EVT1a
EVT1b
EVT1c
EVT2
Fibroblasts
Myofibroblasts
Muscle
Endothelial
Other
VIM
CD31
CD11c
CD3
CD8
CK7
HLA-G
CD57
SMA
CD45
CD56
CD16
DC-SIGN
CD68
HLA-DR
CD14
CD4
CD206
CD163
FOXP3 expression > 0.5
Regulatory T
CD11c expression > 0.5
Mac2b (CD11c+)
Tryptase expression > 0.9
Mast
CD14 expression > 0.5
Located in cell column
Placental Mac
EVT2
Located in cell column?
Yes
No
Glandular

c

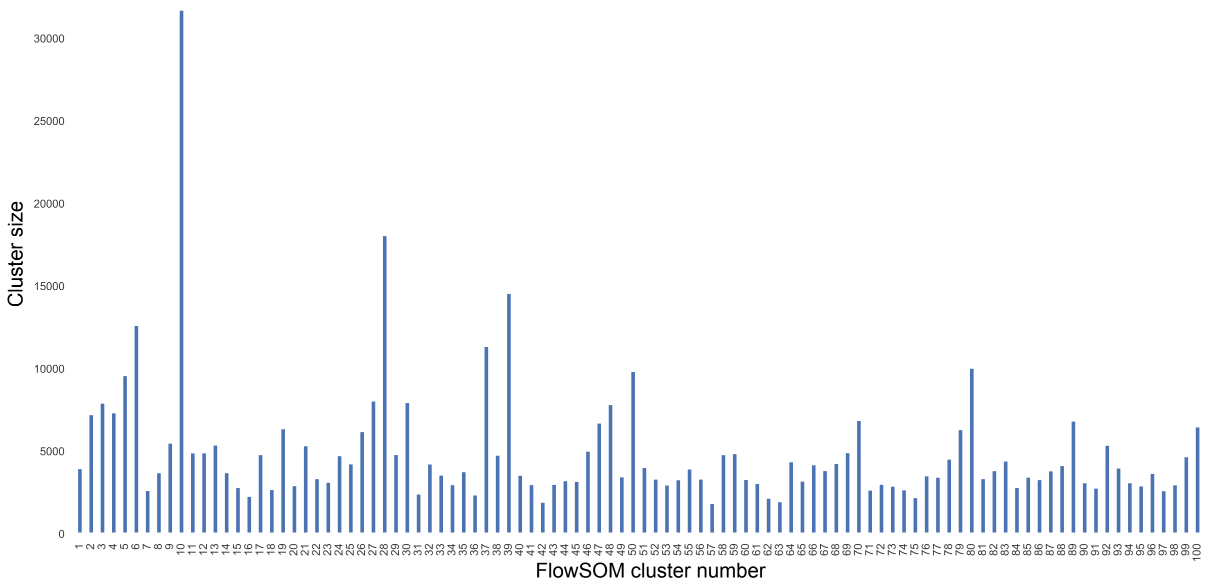
Cluster size
FlowSOM cluster number
0
5000
10000
15000
20000
25000
30000

d

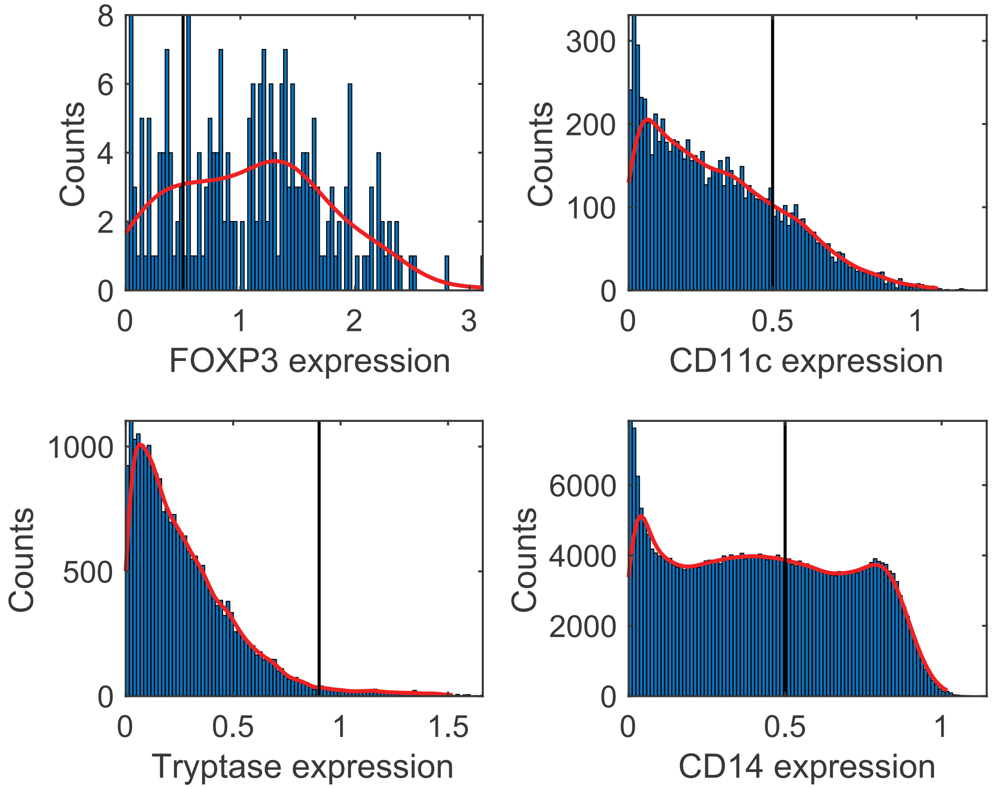
Counts
FOXP3 expression
Counts
CD11c expression
Counts
Tryptase expression
Counts
CD14 expression

e

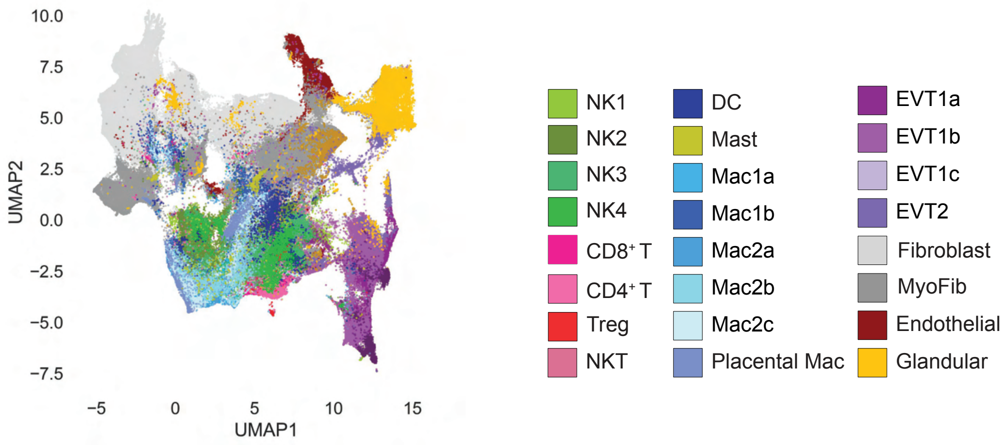
10.0
7.5
5.0
2.5
0.0
−2.5
−5.0
−7.5
UMAP2
−5
0
5
10
15
UMAP1
NK1
NK2
NK3
NK4
CD8⁺ T
CD4⁺ T
Treg
NKT
DC
Mast
Mac1a
Mac1b
Mac2a
Mac2b
Mac2c
Placental Mac
EVT1a
EVT1b
EVT1c
EVT2
Fibroblast
MyoFib
Endothelial
Glandular

f

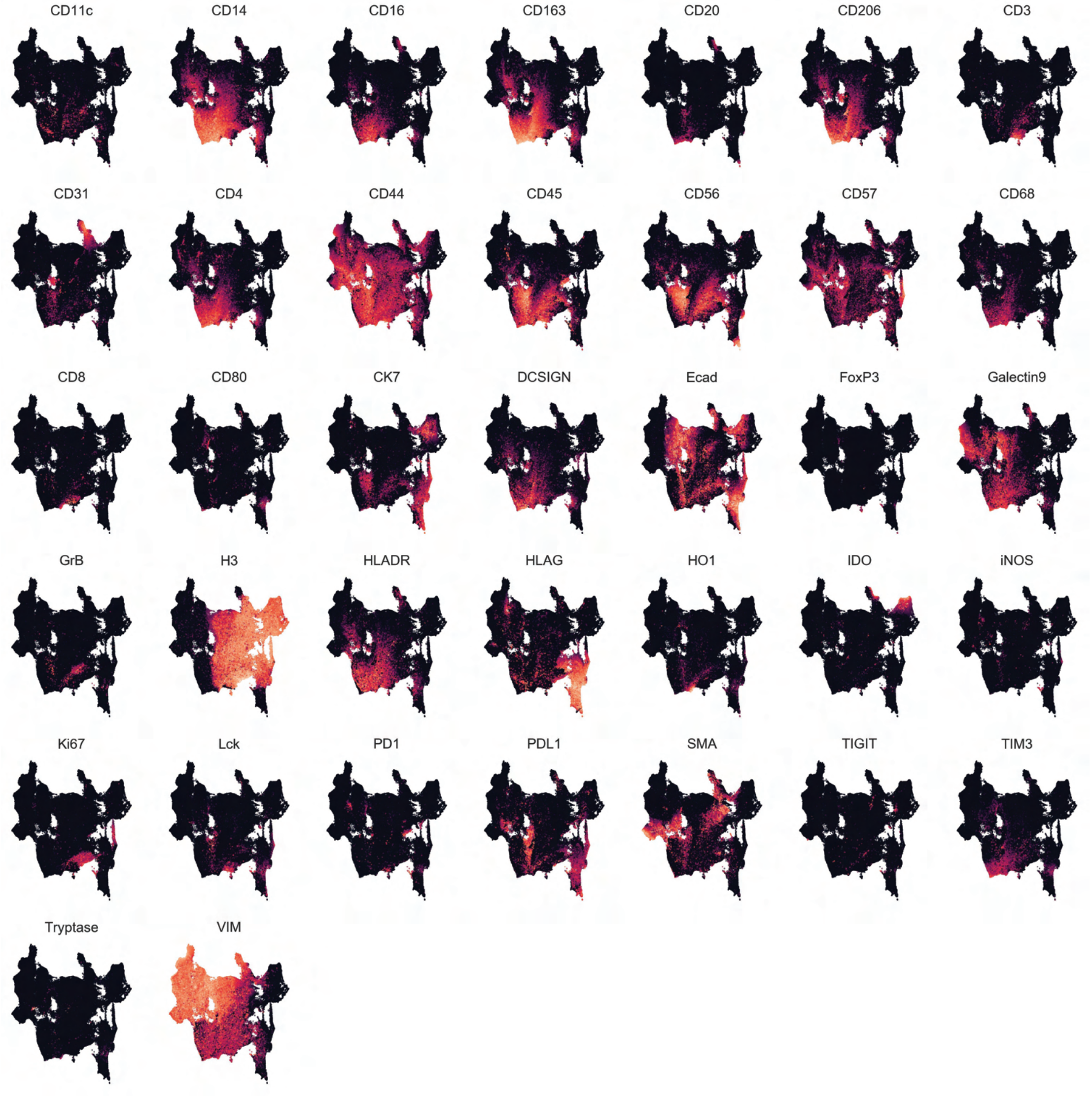
CD11c
CD14
CD16
CD163
CD20
CD206
CD3
CD31
CD4
CD44
CD45
CD56
CD57
CD68
CD8
CD80
CK7
DCSIGN
Ecad
FoxP3
Galectin9
GrB
H3
HLADR
HLAG
HO1
IDO
iNOS
Ki67
Lck
PD1
PDL1
SMA
TIGIT
TIM3
Tryptase
VIM

g

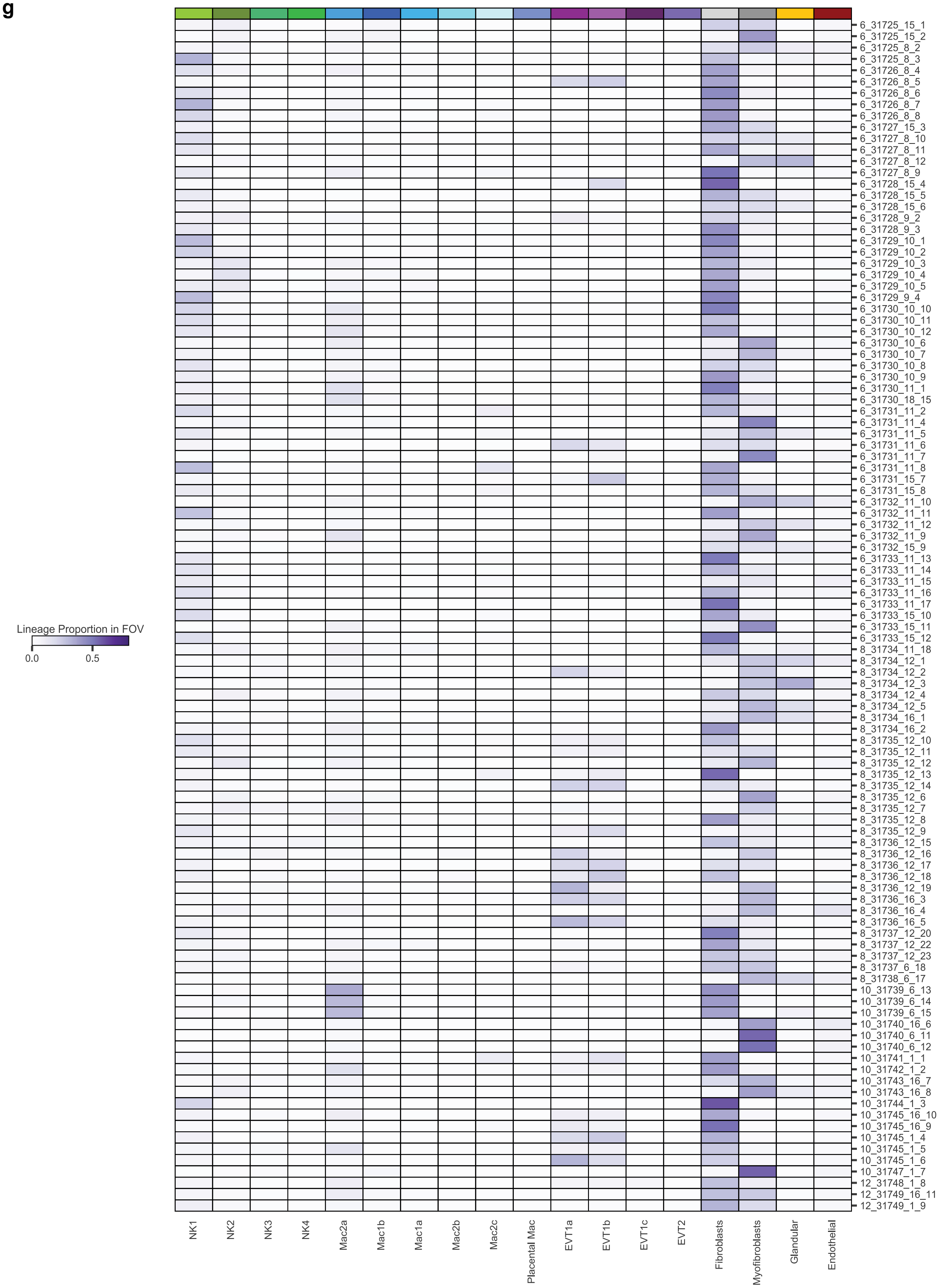
Lineage Proportion in FOV
0.0
0.5
NK1
NK2
NK3
NK4
Mac2a
Mac1b
Mac1a
Mac2b
Mac2c
Placental Mac
EVT1a
EVT1b
EVT1c
EVT2
Fibroblasts
Myofibroblasts
Glandular
Endothelial
6_31725_15_1
6_31725_15_2
6_31725_8_2
6_31725_8_3
6_31726_8_4
6_31726_8_5
6_31726_8_6
6_31726_8_7
6_31726_8_8
6_31727_15_3
6_31727_8_10
6_31727_8_11
6_31727_8_12
6_31727_8_9
6_31728_15_4
6_31728_15_5
6_31728_15_6
6_31728_9_2
6_31728_9_3
6_31729_10_1
6_31729_10_2
6_31729_10_3
6_31729_10_4
6_31729_10_5
6_31729_9_4
6_31730_10_10
6_31730_10_11
6_31730_10_12
6_31730_10_6
6_31730_10_7
6_31730_10_8
6_31730_10_9
6_31730_11_1
6_31730_18_15
6_31731_11_2
6_31731_11_4
6_31731_11_5
6_31731_11_6
6_31731_11_7
6_31731_11_8
6_31731_15_7
6_31731_15_8
6_31732_11_10
6_31732_11_11
6_31732_11_12
6_31732_11_9
6_31732_15_9
6_31733_11_13
6_31733_11_14
6_31733_11_15
6_31733_11_16
6_31733_11_17
6_31733_15_10
6_31733_15_11
6_31733_15_12
8_31734_11_18
8_31734_12_1
8_31734_12_2
8_31734_12_3
8_31734_12_4
8_31734_12_5
8_31734_16_1
8_31734_16_2
8_31735_12_10
8_31735_12_11
8_31735_12_12
8_31735_12_13
8_31735_12_14
8_31735_12_6
8_31735_12_7
8_31735_12_8
8_31735_12_9
8_31736_12_15
8_31736_12_16
8_31736_12_17
8_31736_12_18
8_31736_12_19
8_31736_16_3
8_31736_16_4
8_31736_16_5
8_31737_12_20
8_31737_12_22
8_31737_12_23
8_31737_6_18
8_31738_6_17
10_31739_6_13
10_31739_6_14
10_31739_6_15
10_31740_16_6
10_31740_6_11
10_31740_6_12
10_31741_1_1
10_31742_1_2
10_31743_16_7
10_31743_16_8
10_31744_1_3
10_31745_16_10
10_31745_16_9
10_31745_1_4
10_31745_1_5
10_31745_1_6
10_31747_1_7
12_31748_1_8
12_31749_16_11
12_31749_1_9

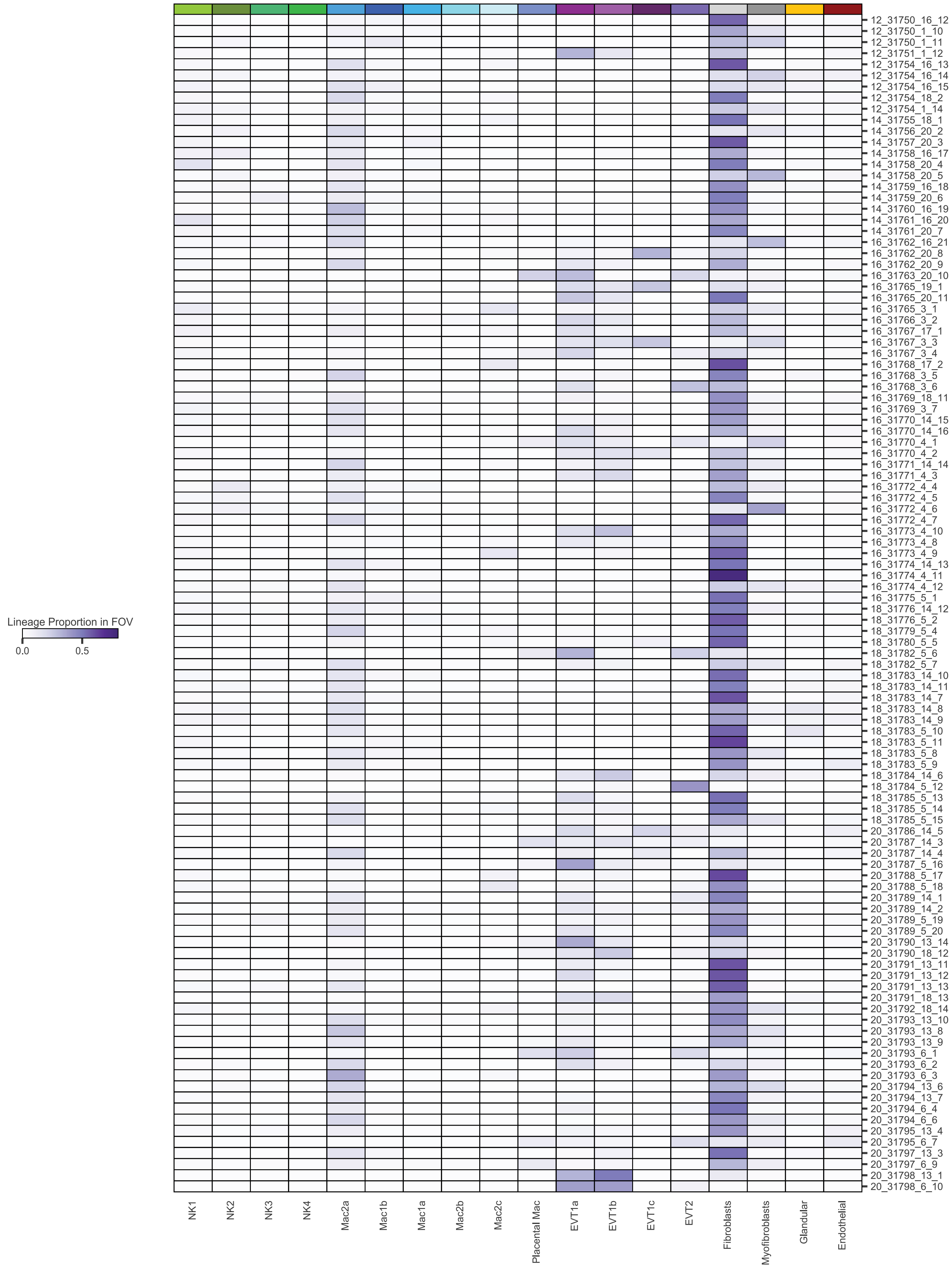
Lineage Proportion in FOV
0.0
0.5
12_31750_16_12
12_31750_1_10
12_31750_1_11
12_31751_1_12
12_31754_16_13
12_31754_16_14
12_31754_16_15
12_31754_18_2
12_31754_1_14
14_31755_18_1
14_31756_20_2
14_31757_20_3
14_31758_16_17
14_31758_20_4
14_31758_20_5
14_31759_16_18
14_31759_20_6
14_31760_16_19
14_31761_16_20
14_31761_20_7
16_31762_16_21
16_31762_20_8
16_31762_20_9
16_31763_20_10
16_31765_19_1
16_31765_20_11
16_31765_3_1
16_31766_3_2
16_31767_17_1
16_31767_3_3
16_31767_3_4
16_31768_17_2
16_31768_3_5
16_31768_3_6
16_31769_18_11
16_31769_3_7
16_31770_14_15
16_31770_14_16
16_31770_4_1
16_31770_4_2
16_31771_14_14
16_31771_4_3
16_31772_4_4
16_31772_4_5
16_31772_4_6
16_31772_4_7
16_31773_4_10
16_31773_4_8
16_31773_4_9
16_31774_14_13
16_31774_4_11
16_31774_4_12
16_31775_5_1
18_31776_14_12
18_31776_5_2
18_31779_5_4
18_31780_5_5
18_31782_5_6
18_31782_5_7
18_31783_14_10
18_31783_14_11
18_31783_14_7
18_31783_14_8
18_31783_14_9
18_31783_5_10
18_31783_5_11
18_31783_5_8
18_31783_5_9
18_31784_14_6
18_31784_5_12
18_31785_5_13
18_31785_5_14
18_31785_5_15
20_31786_14_5
20_31787_14_3
20_31787_14_4
20_31787_5_16
20_31788_5_17
20_31788_5_18
20_31789_14_1
20_31789_14_2
20_31789_5_19
20_31789_5_20
20_31790_13_14
20_31790_18_12
20_31791_13_11
20_31791_13_12
20_31791_13_13
20_31791_18_13
20_31792_18_14
20_31793_13_10
20_31793_13_8
20_31793_13_9
20_31793_6_1
20_31793_6_2
20_31793_6_3
20_31794_13_6
20_31794_13_7
20_31794_6_4
20_31794_6_6
20_31795_13_4
20_31795_6_7
20_31797_13_3
20_31797_6_9
20_31798_13_1
20_31798_6_10
NK1
NK2
NK3
NK4
Mac2a
Mac1b
Mac1a
Mac2b
Mac2c
Placental Mac
EVT1a
EVT1b
EVT1c
EVT2
Fibroblasts
Myofibroblasts
Glandular
Endothelial

h

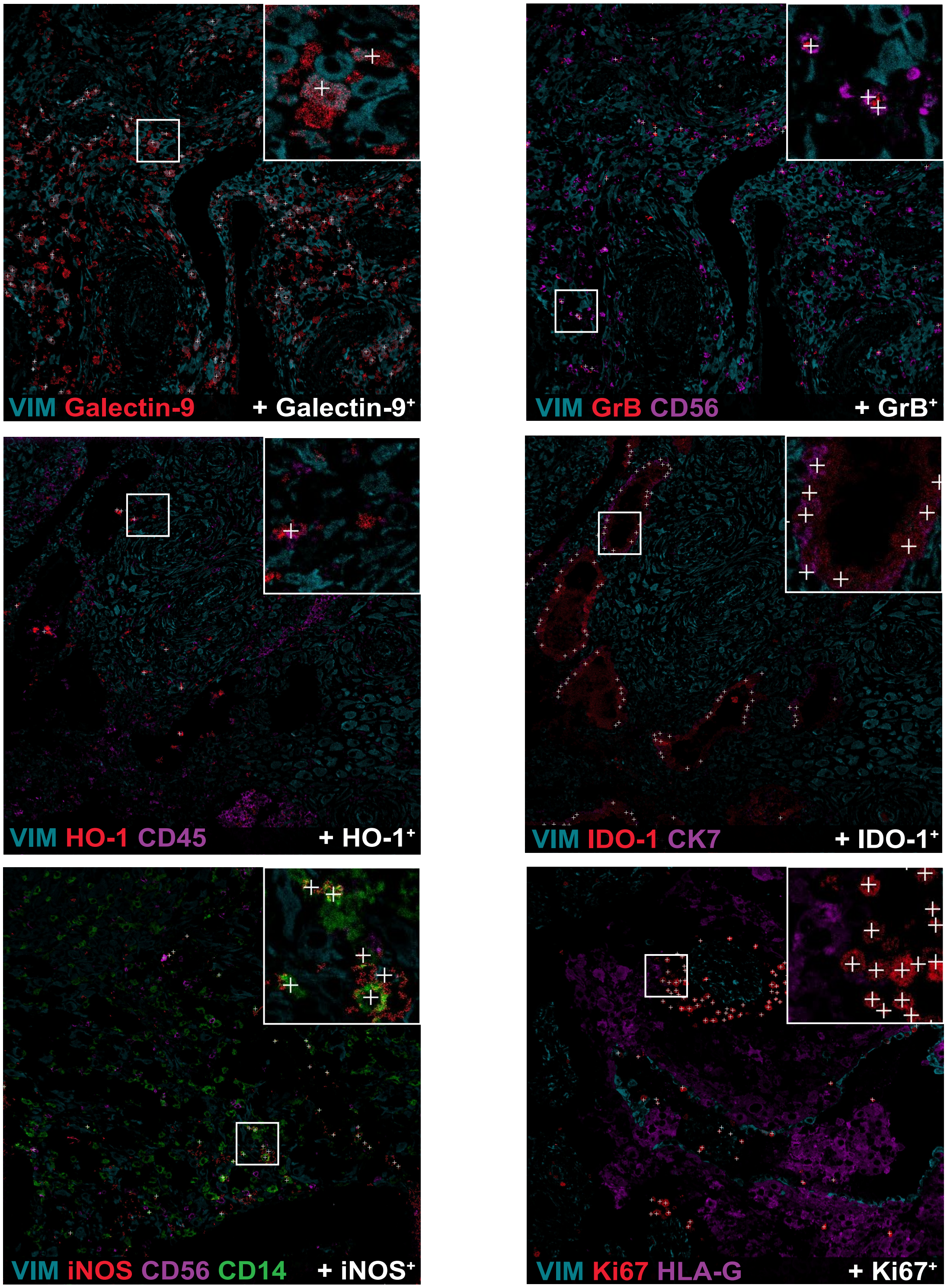
VIM Galectin-9
+ Galectin-9⁺
VIM GrB CD56
+ GrB⁺
VIM HO-1 CD45
+ HO-1⁺
VIM IDO-1 CK7
+ IDO-1⁺
VIM iNOS CD56 CD14
+ iNOS⁺
VIM Ki67 HLA-G
+ Ki67⁺

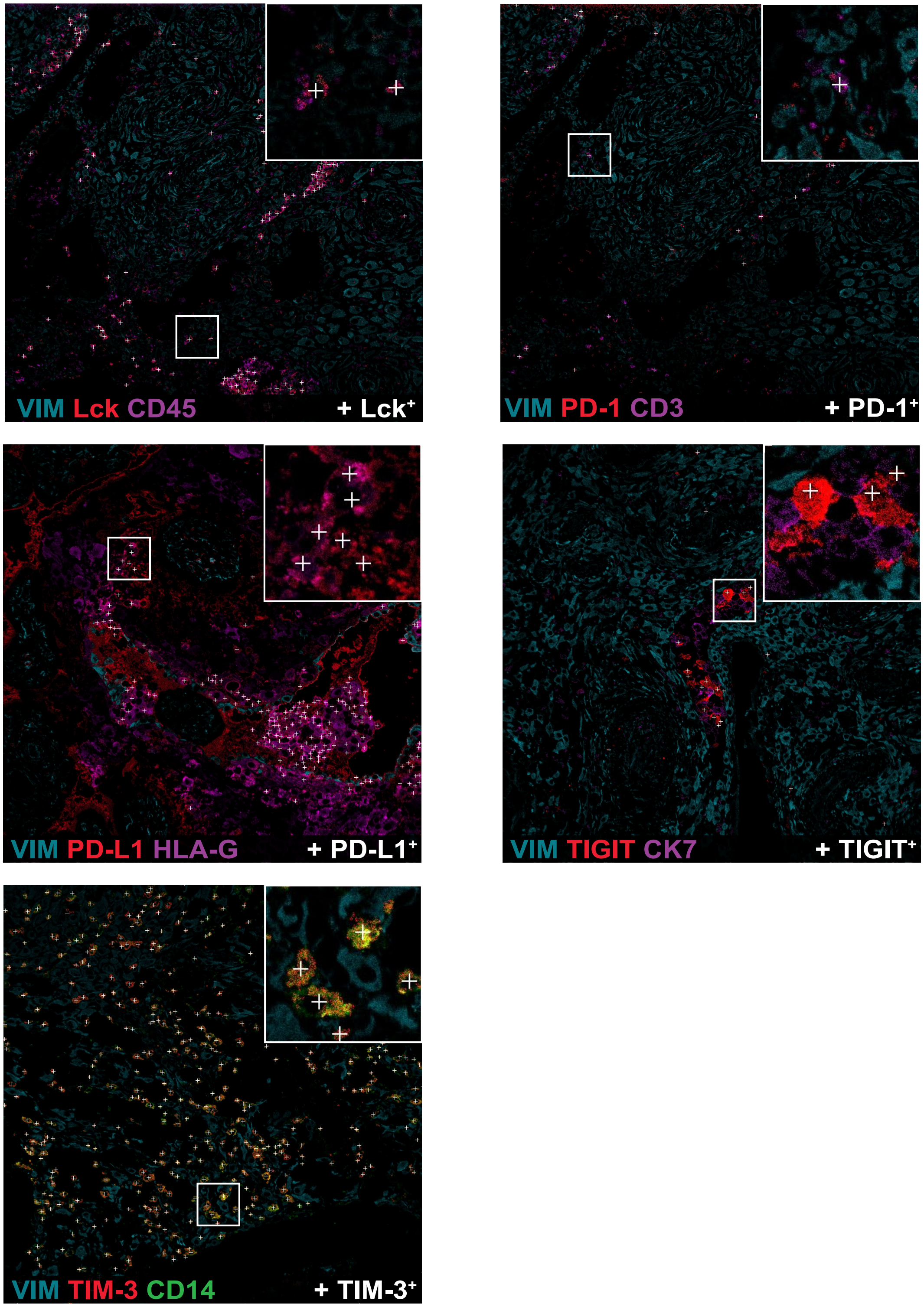

Supplementary Information Figure 2 | Workflow for Mesmer whole-cell segmentation of single cells from MIBI images and cell phenotyping. a. Workflow. (1) Input of manually annotated decidua MIBI images to train our custom convolutional neural network (CNN) Mesmer. (2) Six-channel inputs to Mesmer and outputs (probability maps for cell shape and the center of each cell) with subsequent watershed transformation producing labeled, segmented images.

(3) Post-processing parameters used to more accurately represent marker expression by cell populations located in the glands and anchoring (cell column) villi. VIM, vimentin. (4) Pseudocolored MIBI overlay showing final whole-cell segmentation of a representative MIBI image. b. Overview of steps involved in single-cell phenotyping. (Left to right) Output from FlowSOM (100 clusters) and subsequent assignment to cell phenotypes..c. Number of cells assigned to each FlowSOM cluster d. Histograms of normalized marker expression distributions for FOXP3, CD11c, tryptase, and CD14, with thresholds (vertical line) used for cell phenotyping. e. UMAP of dataset with cell phenotype assignments overlaid. f. UMAPs of dataset with marker expression overlaid. g. Heatmap showing proportion of cells belonging to each assigned lineage in each FOV. h. Representative MIBI overlays showing positive functional marker expression. White crosses indicate cells positive for each functional marker according to the set binary threshold. Representative image of n=3 FOVs per marker.

Analysis of NanoString Whole Transcriptome Data for Decidua ROIs

For this analysis on decidua samples, only genes with background subtracted, normalized counts ≥10 in at least two decidua samples were considered. This resulted in 13104 expressed genes (see Methods "Normalization and scaling of GeoMx counts data").

To estimate the contribution of cell type frequencies in decidua samples to the observed changes in gene expression, cell-type abundancies were estimated in decidua ROIs using the online version of the deconvolution algorithm CIBERSORTx. A custom signature matrix was created using the droplet-based 10x single-cell transcriptome profiles from 30 maternal-fetal tissue samples from Vento-Tormo et al (dataset: https://www.ebi.ac.uk/biostudies/arrayexpress/studies/E-MTAB-6701#). Only cell annotations originating from decidua and placenta were used in creating the signature matrix. This signature matrix was used to impute cell fractions on the Nanostring decidua ROIs (Supplementary Table 13).
We then compared the cell type frequencies imputed by CIBERSORTx to the cell type frequencies we measured by MIBI in the decidua masks of the matched MIBI images. We limited this analysis to cell types that are abundant enough in decidua based on our MIBI measurements (frequency ≥1%). In order to match cell type annotations between the two different data sets, we grouped the cell types from the Vento-Tormo et al. (Nature, 2018) dataset and matched them with MIBI as specified in Supplementary Table 13.
We then leveraged the deconvolved cell frequencies to predict which genes are trending with gestational age in decidual samples and which trends are better described by tissue composition or SAR. To do that, for each gene, we performed the following analysis:

1. Perform separate polynomial regressions (same as analysis described for artery samples in Methods) of gene expression as a function of gestational age, SAR and each of the six cell type frequencies described in Supplementary Table 13
2. Considering only regression models with p-values ≤ 0.05 and fold change ≥ 2, rank models by their R^2
3. Classify the gene as trending with gestational age, SAR or with one of the cell frequencies based on the model with maximal R^2
4. If there were no regression models with p-values ≤ 0.05 and fold change ≥ 2 for the gene, classify it as non-trending

For Coordinated gene expression by pathways in decidua samples we considered all genes for which GA trend was significantly better than SAR trend regardless of tissue composition trends (Supplementary Information Figure 3). Analysis was performed the

same way as described for arteries in Methods section “Coordinated gene expression by pathways in artery”.

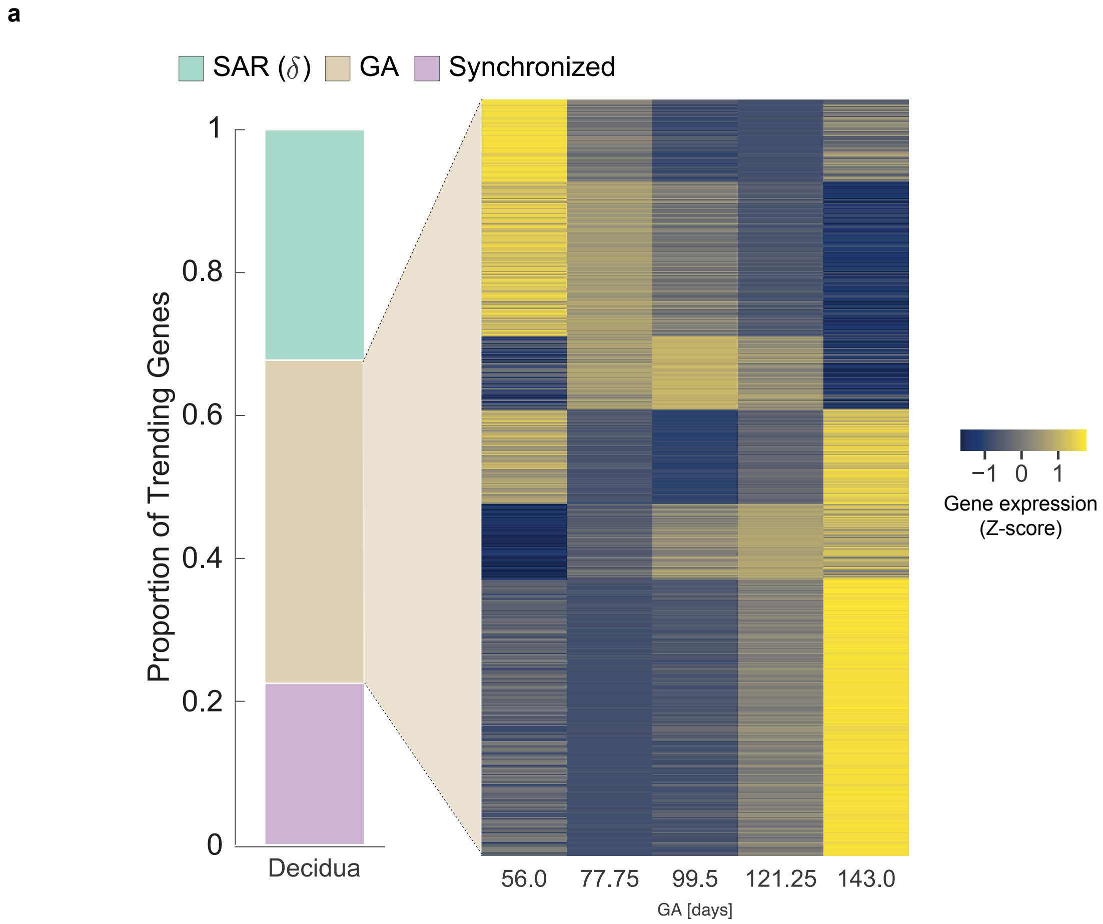

Supplementary Information Figure 3 | Decidual ROIs Nanostring data analysis. a. *Proportion of genes in decidua (1722 total) tissue where expression changes significantly correlate with GA (778), SAR (δ) (555), or both (389). Heatmap inset: GA-correlated genes in decidua showing mean normalized expression (Z-score) by binned GA. Genes shown are only those most correlated with GA after comparing each gene against GA and SAR.*

Tim-3 and Galectin-9 Co-expression in single cells

Tim-3 and Galectin-9 coordinated up-regulation was observed within single cells for decidual macrophages (Supplementary Information Figure 4).

a

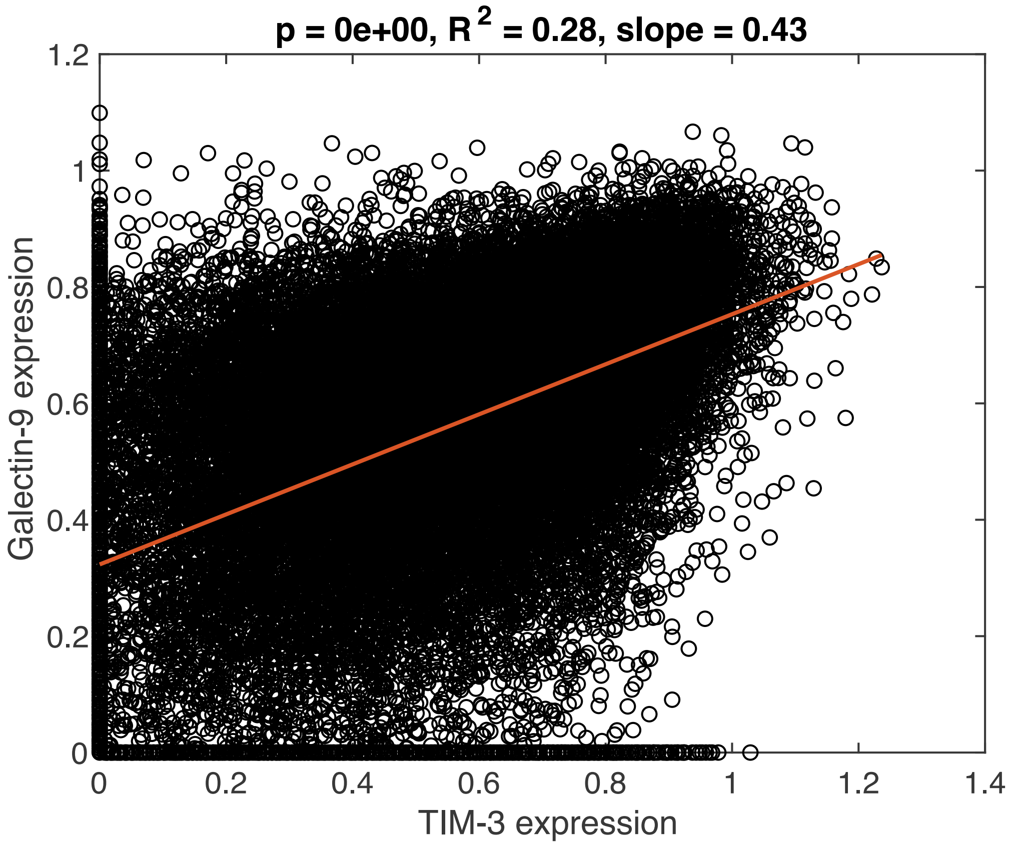

Supplementary Information Figure 4 | Single-cell expression of immunoregulatory markers. a. *Source data: Scatter plot of the expression of TIM-3 and galectin-9 in individual Mac2a and Mac2b cells from all images. Red line indicates fitted linear regression model.*
