# Supplementary material for: A spatially resolved timeline of the human maternal–fetal interface
Source: Nature. 2023 Jul 19;619(7970):595–605. doi: 10.1038/s41586-023-06298-9 (PMC10356615; doi:10.1038/s41586-023-06298-9)
Supplement: Supplementary file 2 — Reporting Summary [file 41586_2023_6298_MOESM2_ESM.pdf]

nature portfolio

Corresponding author(s): Shirley Greenbaum
Michael Angelo

Last updated by author(s): November 4 2022

Reporting Summary

Nature Portfolio wishes to improve the reproducibility of the work that we publish. This form provides structure for consistency and transparency in reporting. For further information on Nature Portfolio policies, see our Editorial Policies and the Editorial Policy Checklist.

Statistics

For all statistical analyses, confirm that the following items are present in the figure legend, table legend, main text, or Methods section.

n/a	Confirmed	
☐	☒	The exact sample size (*n*) for each experimental group/condition, given as a discrete number and unit of measurement
☐	☒	A statement on whether measurements were taken from distinct samples or whether the same sample was measured repeatedly
☐	☒	The statistical test(s) used AND whether they are one- or two-sided<br>*Only common tests should be described solely by name; describe more complex techniques in the Methods section.*
☐	☒	A description of all covariates tested
☐	☒	A description of any assumptions or corrections, such as tests of normality and adjustment for multiple comparisons
☐	☒	A full description of the statistical parameters including central tendency (e.g. means) or other basic estimates (e.g. regression coefficient) AND variation (e.g. standard deviation) or associated estimates of uncertainty (e.g. confidence intervals)
☐	☒	For null hypothesis testing, the test statistic (e.g. *F*, *t*, *r*) with confidence intervals, effect sizes, degrees of freedom and *P* value noted<br>*Give P values as exact values whenever suitable.*
☒	☐	For Bayesian analysis, information on the choice of priors and Markov chain Monte Carlo settings
☒	☐	For hierarchical and complex designs, identification of the appropriate level for tests and full reporting of outcomes
☒	☐	Estimates of effect sizes (e.g. Cohen's *d*, Pearson's *r*), indicating how they were calculated

Our web collection on statistics for biologists contains articles on many of the points above.

Software and code

Policy information about availability of computer code

Data collection	For clinical patient data: Electronic Medical Record– ('eCW' - electronic clinical works) or handwritten forms
Data analysis	Software used for analysis in the paper: ImageJ, MAUI (https://github.com/angelolab/MAUI) for low level image processing, DeepCell version 0.5.0 (https://deepcell.readthedocs.io/en/master/index.html) for cell segmentation. Ark Analysis for cell-cell spatial enrichment (https://github.com/angelolab/ark-analysis). QuPath (Version: 0.4.0) for two color IHC analysis. Custom code for this study is available at doi:10.6084/m9.figshare.16663465.

For manuscripts utilizing custom algorithms or software that are central to the research but not yet described in published literature, software must be made available to editors and reviewers. We strongly encourage code deposition in a community repository (e.g. GitHub). See the Nature Portfolio guidelines for submitting code & software for further information.

Data

Policy information about availability of data

All manuscripts must include a data availability statement. This statement should provide the following information, where applicable:

- Accession codes, unique identifiers, or web links for publicly available datasets
- A description of any restrictions on data availability
- For clinical datasets or third party data, please ensure that the statement adheres to our policy

MIBI imaging data is available at https://doi.org/10.35079/hbm585.qpdv.454. Same MIBI imaging data in a browsable format, along with segmentation masks, extracted features, CPMs, cell-cell and cell-artery spatial enrichment scores per image, a table enumerating all single cells in this study and provides their location, morphological characteristics (such as size and shape), marker expression, FlowSOM cluster assignment and cell type assignment are available at doi:10.6084/m9.figshare.16663465. Patient block H&E images with annotations are available at https://doi.org/doi:10.5061/dryad.v15dv41zp. The following public databases

were used in this study (see Methods for details): Biological Process (GO-BP) database (http://geneontology.org/), "Gene Cards" database (https://www.genecards.org/).

Field-specific reporting

Please select the one below that is the best fit for your research. If you are not sure, read the appropriate sections before making your selection.

☒ Life sciences ☐ Behavioural & social sciences ☐ Ecological, evolutionary & environmental sciences

For a reference copy of the document with all sections, see nature.com/documents/nr-reporting-summary-flat.pdf

Life sciences study design

All studies must disclose on these points even when the disclosure is negative.

Sample size	Cohort consisted of archival tissue from the decidua of 71 patients. While no statistical methods were used to determine cohort size, this is the largest to date cohort used for the study of the human maternal-fetal interface and is therefore sufficient.
Data exclusions	Patient samples were screened by a pathologist and samples not containing decidua were excluded. Exclusion criteria of not including non decidual samples were pre-established and 66 patients remained after exclusion.
Replication	Experimental findings were based on 66 patients, several images were generated per patient.
Randomization	Irrelevant since there were no experimental groups in this study.
Blinding	Blinding was irrelevant due to the absence of experimental groups.

Reporting for specific materials, systems and methods

We require information from authors about some types of materials, experimental systems and methods used in many studies. Here, indicate whether each material, system or method listed is relevant to your study. If you are not sure if a list item applies to your research, read the appropriate section before selecting a response.

Materials & experimental systems

n/a	Involved in the study	
☐	☒	Antibodies
☒	☐	Eukaryotic cell lines
☒	☐	Palaeontology and archaeology
☒	☐	Animals and other organisms
☐	☒	Human research participants
☒	☐	Clinical data
☒	☐	Dual use research of concern

Methods

n/a	Involved in the study	
☒	☐	ChIP-seq
☒	☐	Flow cytometry
☒	☐	MRI-based neuroimaging

Antibodies

Antibodies used	Study used 38 primary antibodies. See Supplemental data table 9 for all requested information about each reagent including the precise working concentration used (called "Titer").
Validation	All reagents were validated in-house with chromogenic immunohistochemistry on FFPE human control tissue. Following this stage of validation all antobodies were metal-labeled and further tested with MIBI-TOF. Imaging data for all antibodies in control tissues can be found in Extended Data Figure 1.

Human research participants

Policy information about studies involving human research participants

Population characteristics	Study did not directly involve human participants, but used archival cloinical specimens from the decudua of 66 patients who underwent elective pregnancy termination between 6-20 weeks gestation at San Francisco General Hospital. Patients were aged 20-39, with parity of 0-4, racially diverse (While, Hispanic, Asian, Black) and with BMI ranging 19-48. Additional information about the patients appears in Supplementary table 1.
Recruitment	No participants were recruited, archival tissue was used. Per protocol #46646 from Stanford IRB board, consent to use archival deidentified tissue was not required
Ethics oversight	All human samples were acquired in accordance with Institutional Review Board (IRB) protocol #46646 "Assessing Normal

Ethics oversight	Expression Patterns of Immune and Non-Immune Markers Across Tissue Types With Mulitplexed Ion Beam Imaging" at Stanford University.

Note that full information on the approval of the study protocol must also be provided in the manuscript.
